# Fungal and bacterial communities and their associations in snow-free and snow covered (sub-)alpine *Pinus cembra* forest soils

**DOI:** 10.1186/s40793-024-00564-7

**Published:** 2024-04-02

**Authors:** Maraike Probst, Anusha Telagathoti, Edoardo Mandolini, Ursula Peintner

**Affiliations:** Department for Microbiology, Universität Innsbruck, Technikerstrasse 25, 6020 Innsbruck, Austria

**Keywords:** Microbial association networks, Microbial interaction, Mountain forest soil, Mycorrhiza, Seasonality

## Abstract

**Background:**

In Europe, *Pinus cembra* forests cover subalpine and alpine areas and they are of high conservational and ecological relevance. These forests experience strong seasonality with alternating snow-free and snow covered periods. Although *P. cembra* is known for mycorrhization and mycorrhizae usually involve fungi, plants and bacteria, the community compositions of fungi and bacteria and their associations in (sub-)alpine *P. cembra* forests remain vastly understudied. Here, we studied the fungal and bacterial community compositions in three independent (sub-)alpine *P. cembra* forests and inferred their microbial associations using marker gene sequencing and network analysis. We asked about the effect of snow cover on microbial compositions and associations. In addition, we propose inferring microbial associations across a range of filtering criteria, based on which we infer well justified, concrete microbial associations with high potential for ecological relevance that are typical for *P. cembra* forests and depending on snow cover.

**Results:**

The overall fungal and bacterial community structure was comparable with regards to both forest locations and snow cover. However, occurrence, abundance, and diversity patterns of several microbial taxa typical for *P. cembra* forests differed among snow-free and snow covered soils, e.g. *Russula, Tetracladium* and *Phenoliphera.* Moreover, network properties and microbial associations were influenced by snow cover. Here, we present concrete microbial associations on genus and species level that were repeatedly found across microbial networks, thereby confirming their ecological relevance. Most importantly, ectomycorrhizal fungi, such as *Basidioascus, Pseudotomentella* and *Rhizopogon,* as well as saprobic *Mortierella* changed their bacterial association partners depending on snow cover.

**Conclusion:**

This is the first study researching fungal-bacterial associations across several (sub-)alpine *P. cembra* forests. The poorly investigated influence of snow cover on soil fungi and bacteria, especially those mycorrhizing *P. cembra* roots, but also saprobic soil organisms, underlines the relevance of forest seasonality. Our findings highlight that the seasonal impact of snow cover has significant consequences for the ecology of the ecosystem, particularly in relation to mycorrhization and nutrient cycling. It is imperative to consider such effects for a comprehensive understanding of the functioning resilience and responsiveness of an ecosystem.

**Supplementary Information:**

The online version contains supplementary material available at 10.1186/s40793-024-00564-7.

## Background

Forest ecosystems are crucial for nutrient cycling, oxygen production, and air filtering. Moreover, they prevent soil erosion and they harbour a wide range of species, including fungi and bacteria. For humans, they are valuable for several reasons, such as providing recreational areas and timber. In Europe, *Pinus cembra* forests are common in subalpine and alpine areas. They usually occur between 1500 and 2500 m above sea level (a.s.l.) [1] in both siliceous and calcareous environments. This endemic species is of high conservational value and it can withstand extreme conditions, allowing it to colonize areas other species cannot survive. Due to the complex topography of (sub-)alpine areas, mountain forests usually exhibit high local-scale heterogeneity [2]. In comparison to habitats at lower altitudes, (sub-)alpine habitats undergo strong seasonal changes: In snow-free summer, temperature and UV radiation are high and water is often scarce. In winter, soils are typically snow covered and temperatures remain well below freezing point. In between snow-free and snow covered periods, transitional periods are common. They include freeze–thaw cycles and periods with increased water intake due to precipitation.

Snow cover insulates soil, thereby preventing gas exchange and nutrient intake from aboveground, thus impacting various ecological processes. It influences hydrology [3] and soil nutrient fluxes [4] by increasing soil moisture, C, N, and P content [5]. Under snow cover, temperature remains around 0 °C [6] and it allows for higher microbial biomass [5], soil enzymatic activities, and soil respiration compared to freezing air temperatures [4]. These conditions also shape ecological niches and time the growing season [7]. Furthermore, there are strong legacy effects, with snow cover affecting the conditions and performance of the following growing season [5]. For these (and more) reasons, (sub-)alpine regions are especially sensitive to global warming, thereby highlighting the need for better understanding (sub-)alpine forest ecosystems in order to predict their functioning in future warmer climate scenarios and to prevent their destruction as far as possible.

*P. cembra* roots are usually highly mycorrhized by ectomycorrhizal fungi. Mycorrhizal fungi form an intimate, symbiontic interaction with their plant host, which is of utmost relevance for forest health, as it supplies nutrients and water to the plant, improves plant resistance against abiotic stressors, and provides defence against pathogenic organisms [8]. Generalist fungi, colonizing the roots of several different tree species, and specialist fungi, specific to individual tree species, may colonize *P. cembra* roots [9]. In alpine and subalpine habitats, specialists often dominate the ectomycorrhizal community, possibly, because in high altitude areas with extreme conditions, they provide the benefit of efficient nutrient and water transfer between both symbionts and exclude mycoheterotrophy [10]. Some fungal genera, such as *Suillus, Rhizopogon, Russula,* and *Tomentella,* are well known for mycorrhizing *P. cembra* roots [11].

Although mycorrhiza are usually considered symbiotic interactions among mycorrhizal fungi and plants, a strong point can be made for mycorrhiza being tripartite interactions among mycorrhizal fungi, plants and bacteria [12]. In this case, it may not only be plants and fungi specifically choosing their partner, but fungi influencing the bacterial community assembly, at least those surrounding their hyphae [12]. While some specific interactions are well studied [12], very little is known about these interactions on community level, especially considering seasonal influences [13]. In fact, different microbes are active under the snow compared to those active under snow-free conditions and microbial community structures differ depending on snow cover, as shown for beech [14], oak [15, 16], Scots pine [17], spruce [18], and boreal forest dominated by spruce and pine [19]. As the microbiome is tree-specific [20], and the function carried out depends on the concrete organisms present at that time and under these particular conditions, specific microbial interactions typical for *P. cembra* forests and their variation between snow-free and snow covered conditions need to be explored.

Studying microbial interactions is challenging [21]. Wet-lab experiments offer the advantage of observing direct cell–cell contact and causal inference by specific manipulation. However, such experiments might not resemble natural conditions and neglect complexity. Therefore, they cannot capture ecosystem-level interactions. The combination of molecular and computational approach allows observing a larger part of the microbial community under natural conditions as compared to wet-lab experiments. Although network analysis has been widely applied, it comes with the disadvantage that interactions inferred are associations, which might or might not be ecologically relevant [22]. In addition, different network tools may give different results, and filter criteria, usually applied prior to network inference in order to reduce complexity and increase accuracy, will affect the resulting network [23, 24]. Although there is much room for optimizing current network analysis tools, network inference can contribute to better understanding of the microbial community, its structure and facilitate hypothesis building, thereby promoting future research [22, 25].

Here, we studied the fungal and bacterial communities in snow-free and snow covered soils of three different (sub-)alpine *P. cembra* forests using amplicon sequencing. To our knowledge, this is the first study investigating microbial associations, especially among fungi and bacteria, under snow cover in *P. cembra* forests. We hypothesized that (1) microbial community compositions and fungal-bacterial (fb) association patterns in (sub-)alpine *P. cembra* habitats are comparable independent of their geographical distance; and that (2) microbial communities, network structures, and fb associations differ depending on snow cover, thereby allowing to hypothesize microbial roles in *P. cembra* forests depending on snow cover. We inferred microbial networks across a range of filter criteria in order to obtain robust microbial associations. From a methodological perspective, we asked if associations can be found repeatedly, i.e. independent of filter criteria, thereby increasing the confidence we can have in these associations. From an ecological perspective, we identified the association partners of those microbial units found repeatedly across all *P. cembra* forests studied and microbial units identified as those fungal genera mycorrhizing *P. cembra* roots. From these, we interpreted fb associations typical for *P. cembra* forest and their seasonal dependence.

## Material and methods

### Sampling sites

Soil samples were collected from three different *P. cembra* subalpine and alpine sites located in the Austrian Alps, namely Kühtai, Patscherkofel, and Praxmar (Table 1). All locations sampled were dominated by *P. cembra*; some few *P. abies* specimen were present in Praxmar.

**Table 1:** *Pinus cembra* forests included in the study

Location	Altitude [m a.s.l.]	Habitat	pH		C/N		Moisture [%]		Soil organic matter [% dw]		#Samples		Comment
			Snow-free	Snow covered	Snow-free	Snow covered	Snow-free	Snow covered	Snow-free	Snow covered	Snow-free	Snow covered	
Kühtai	1880–2030	*Pinus cembra*	3.5 ± 0.32	3.6 ± 0.36	21 ± 5.9	25 ± 3.7	60 ± 16	50 ± 13	60 ± 26	50 ± 27	13	12	Three sites total at different altitudes and coordinates: 2030 m a.s.l.; 47.217208, 11.036823 1910 m a.s.l.; 47.214527, 10.991395 1880 m a.s.l.; 47.208363, 11.006565
Patscherkofel	2260	*Pinus cembra*	3.6 ± 0.54	3.7 ± 0.60	23 ± 3.7	26 ± 1.8	70 ± 10	60 ± 7	70 ± 20	70 ± 17	19	14	Four sites total with distances of > 50 m to each other Coordinates: 47.9938158, 11.6629124
Praxmar	1520–1820	*Pinus cembra,* *Picea abies*	3.4 ± 0.50	4.0 ± 0.32	19 ± 6.6	19 ± 5.4	40 ± 16	50 ± 13	40 ± 21	20 ± 16	13	8	Three sites total at different altitudes and coordinates: 1820 m a.s.l.; 47.155964, 11.128367 1780 m a.s.l.; 47.154348, 11.130158 1520 m a.s.l.; 47.162253, 11.139553

All sites are located in the Austrian Alps. Per site between 3 and 10 replicates were collected and used for soil physicochemical analysis. Sample numbers indicated in the table refer to samples used for microbiological analysis

a.s.l., above sea level; SOM, soil organic matter; dw, dry weight

At both Kühtai and Patscherkofel, one site was chosen covering an area of approx. 1 km^2^ of the *P. cembra* forests. At Kühtai, five plots were installed; at Patscherkofel, four plots were installed. Distances between plots exceeded 50 m. Samples were collected within plots with > 1 m distance to each other. At Praxmar, the forest area was more extensive covering an area of approx. 5 km^2^ of forest. Here, three individual sites (= plots) were chosen (> 1 km distant). From each site, several samples with > 1 m distance were collected.

Snow-free soils were collected in the beginning of December (2019), prior to first snow fall. Snow covered samples were collected in May/June (2020). At each site and time point, between 8 and 19 soil samples were collected, depending on the size of the site. A total of 79 samples were analysed (Table 1). For each soil sample, around 200 g of top soil (0–20 cm) were collected. An amount of 500 mg was taken and frozen at − 20 °C for DNA analysis. The rest was homogenized by sieving (2 mm) and used immediately for physicochemical analysis of soil properties. Please note that there was no pooling of soil samples or homogenising of large soil volumes prior to DNA extraction. Therefore, the probability of mixing DNA from organisms that were not close to each other in space is relatively low.

### Soil physicochemical analysis

The soil properties were measured as suggested by [26] (Table 1). Briefly, the pH of the soil samples was measured with a pH meter in 1:5 dilutions in 0.01 M Calcium chloride (CaCl_2_). Moisture content was determined based on the weight loss after overnight incubation at 105 °C. From the dry weight, the carbon and nitrogen content was determined using an organic elemental analysis flash combustion instrument (Thermo Fischer scientific, Austria). The soil organic matter (SOM) content was determined from dry weight based on loss of ignition at 550 °C for 7 h. For each physicochemical property measured, a minimum number of 5 snow-free and 5 snow covered representative soil samples were selected from each site.

### DNA extraction and library preparation

Soil samples stored at − 20 °C until analysis were thawed at 4 °C overnight; then, total environmental DNA was extracted using EZNA DNA extraction kit for soil (Omega Bio-tek, Austria). Three non-template controls were added to DNA extraction. They were carried along all analysis including Illumina sequencing and they were treated exactly as all the samples. After extraction, DNA quality and quantity were checked using agarose gel electrophoresis and Qubit E6150 (Promega, Germany). After dilution to 20 ng µl^−1^, the ITS2 and 16S-V4 region of the fungal and prokaryotic rRNA gene, respectively, were amplified from the total environmental DNA extracted using PCR. For fungi, the ITS2 was amplified and primers gITS7 [27] and ITS4 [28] were used. For prokaryotic 16S-V4 region, primers 515f [29] and 806r [30] were used, thereby following the protocol of the Earth Microbiome Project. Primers (5’-3’) with Illumina adapters attached were used for amplification: gITS7 = CTC TTT CCC TAC ACG ACG CTC TTC CGA TCT GTG ART CAT CGA RTC TTT G; ITS4 = CTG GAG TTC AGA CGT GTG CTC TTC CGA TCT TCC TCC GCT TAT TGA TAT GC; V4fw = CTC TTT CCC TAC ACG ACG CTC TTC CGA TCT GTG YCA GCM GCC GCG GTA A; V4rv = CTG GAG TTC AGA CGT GTG CTC TTC CGA TCT GGA CTA CNV GGG TWT CTA AT.

Q5® Hot Start High-Fidelity 2X Master Mix from NEB was used for amplification according to the manufacturer’s instructions; 1 µl 20 ng µl^−1^ DNA extract was used as template. The PCR amplification conditions were as follows: After initial denaturation (95 °C, 5 min), DNA extracts were amplified via 25 cycles of 45 s at 95 °C denaturation, 30 s at 48 °C (fungi) and 54 °C (bacteria), respectively, annealing, and 90 s 72 °C elongation. Each PCR run finished with 5 min final elongation at 72 °C. The quality and quantity of PCR products were checked on agarose gel (1%). In a second PCR step, Illumina adapters and unique barcodes serving as sample identifier were attached to the PCR products (ITS and 16S). For the second PCR, Q5® Hot Start High-Fidelity 2X Master Mix from NEB was used according to the manufacturer’s instructions. All negative controls were carried along; in addition, non-template controls were added. PCR product template was added to the reactions in similar concentrations for all samples. For negative controls, 5 µl PCR product was used as the concentration was too low to be quantified. The cycling conditions of the second PCR were as follows: Initial denaturation at 95 °C for 5 min; 15 cycles of denaturation (95 °C, 45 s), annealing (56 °C, 45 s), elongation (72 °C, 90 s); final elongation at 72 °C for 10 min. PCR products were cleaned up, checked on agarose gel, quantified using Qubit, and pooled in equimolar amounts to obtain the ITS and 16S sequencing library, respectively. In the negative controls, the DNA concentration was below the detection limit. Therefore, 20 µl of each negative control’s cleaned up PCR product was added to the library. Sequencing was carried out on an Illumina MiSeq instrument at Microsynth AG, Switzerland, using two flow cells, one for the ITS and one for the 16S library.

### Bioinformatic analysis

Demultiplexed sequences trimmed off adapters were obtained from Microsynth AG, Switzerland. Sequences had a minimum quality phred score of 20. Sequences were trimmed off the primers using cutadapt (v1.18) [31]. Sequences were deposited in the NCBI SRA under project number PRJNA1005801. In order to obtain an amplicon sequence variant (ASV) abundance table, sequences were further processed using dada2 v1.16.0 [32]. Briefly, sequences were filtered using default settings, i.e. removing ambiguous bases (maxN = 0). The quality of remaining sequences was double-checked using quality plots. Error profiles were predicted from random samples and forward and reverse sequence compositions of the samples were inferred. Forward and reverse sequences were merged. Chimeric sequences were removed from the dataset. In both, the fungal and the bacterial dataset, the percentage of sequences marked as chimeric was low (< 1%). ASVs were inferred using per-sample inference (pool = FALSE (default)). Taxonomic annotation of fungal and bacterial ASVs was performed via dada2 using UNITE 8.3 [33] and Silva 138 [34], respectively. On average, each sample contained 6,500 ± 2,320 fungal and 60,000 ± 16,000 bacterial sequences (average ± standard deviation). As fungal ITS sequences are hypervariable and might considerably vary in their length, no length filtering was applied for fungal ASVs. As the longest fungal ASV sequence was 449 bp, there was sufficient overlap of forward and reverse read for all ASVs (Additional file 1: Fig. S1A). Therefore, all fungal ASVs were kept in the dataset. For bacterial ASVs, ASVs with a sequence > 259 bp and < 247 bp were removed (Additional file 1: Fig. S1B). In all samples, rarefaction curves reached saturation for the fungal and the bacterial dataset, respectively (Additional file 1: Fig. S2). ASV abundances per sample were summarized in a fungal and a bacterial ASV table, respectively. ASV tables were used for all further analysis. All analyses were performed in R 4.0.2 [35].

### Statistical data analysis

All analyses were performed in R 4.0.2 [35]. Scripts from which all data analysis can be reproduced can be found on Github: Maraikep/Pinus.

The influence of snow cover (snow-free vs. snow covered) and location (Kühtai, Patscherkofel, Praxmar) on the fungal and bacterial community composition, respectively, was tested using permutational analysis of variance (Adonis2) [36] as implemented in the package vegan (v. 2.6–4) [37].

The ASV richness and Shannon diversity index of the samples was calculated using vegan [37]. The influence of snow cover and location on the sequencing depth (number of sequences per sample), ASV richness, and Shannon diversity index were compared using Kruskal test. Rarefication of the dataset did not change the interpretation of the data as shown by comparison of alpha diversity measures (Additional file 2: Table S1) and Procrustes analysis on non-metric multidimensional scaling (NMDS) based on Bray Curtis distances (Additional file 1: Fig. S3) from package vegan. Therefore, unrarefied results were reported.

Core ASVs were defined as those ASVs detected in at least one sample from every location (Kühtai, Patscherkofel, Praxmar). Being present in all *P. cembra* forests studied here, we considered these core ASVs as typical for *P. cembra* forests in (sub-)alpine habitats. In order to visualize the differential abundance of core ASVs, a heatmap was drawn using gplots (v3.1.1) [38]. In addition, we calculated the average ASVs’ relative abundances within each sample group (location*snow cover) and looked for ASVs that were clearly of high relative abundance compared to all other ASVs within the sample group. These abundant ASVs within sample groups, we compared (i) across sample groups and (ii) to the core ASVs in order to check for patterns according to experimental factors.

**Fig. 1:**
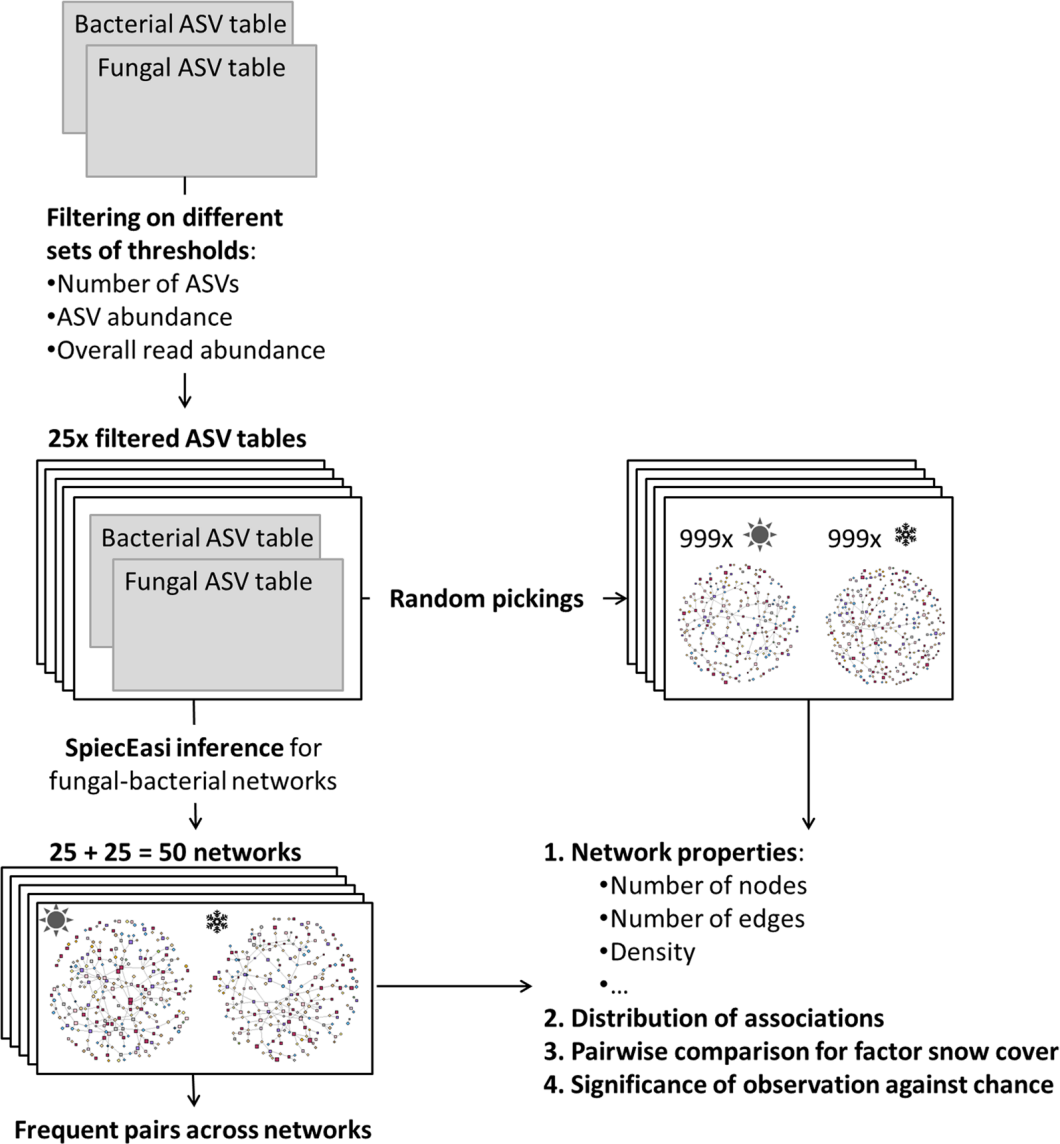
Schematic overview of the network inference procedure and the statistical comparison of the networks inferred

In order to analyse the associations among fungi and bacteria, fungal-fungal, fb and bacterial-bacterial association networks were inferred using SpiecEasi (v1.1.1) [39]. As for this analysis, two datasets (fungi and bacteria) needed to be analysed jointly, we used the multi.spiec.easi command (method = "mb", sel.criterion = "stars", verbose = T, pulsar.select = T). This command is specially designed to combine two datasets, thereby taking care of unbiased centred log-ratio calculation of the joint dataset. This study had two main aims, namely (i) fungal and bacterial associations, especially fb associations, in *P. cembra* forest soils and (ii) differences depending on snow cover. Therefore, and as no differences were detected in microbial composition among locations, networks were calculated across all locations. One network was inferred from snow-free and one from snow covered samples, with each network including the samples from all locations. We applied a range of filtering criteria to the fungal and bacterial ASV table, respectively, and inferred sets of snow-free and snow covered networks from the filtered datasets (Fig. 1). This was done for two reasons: First, two networks can be described in a comparative manner but they cannot be compared statistically. Second, associations inferred by network prediction depend on the input dataset and rare ASVs, including commonly applied, artificial thresholds for removing rare ASVs, can influence the associations inferred [22, 23, 39]. The sets of networks across filtering criteria help overcoming these problems as they provide network replicates, which allow for statistical comparison of network properties and more robust inference of microbial associations (Fig. 1). For justification and discussion of pros and cons of this procedure, please see the last part of the discussion.

We applied systematic filtering criteria to the ASV tables, which were based on the frequency of ASV occurrences across samples, rather than their abundance. This involved removing ASVs detected in fewer than a predetermined number of samples, a threshold determined by examining the ASV distribution and considering the total number and size of ASVs retained in the dataset (Additional file 2: Tables S2, S3). Thresholds were selected via commonly used shoulder theory based on the distribution of one or more of these characteristics; i.e. drops in the distributions of read abundance in the overall dataset, number of ASVs, ASV abundances (Additional file 2: Tables S2, S3). For the fungal ASV table, thresholds of 5, 7, 9, 11, and 19 samples were set. For the bacterial ASV table, thresholds of 10, 20, 31, 39, and 46 samples were set. By combining these thresholds, a number of 5 × 5 = 25 filtered ASV tables were generated. From each filtered ASV table, a snow-free and a snow covered network were inferred, resulting in 25 + 25 = 50 networks total. For all networks, orphaned nodes, i.e. ASVs not connected to any other ASV, were removed as they do not add information on microbial associations. For each of these networks, the following network properties were analysed: number of both bacterial and fungal ASVs in the snow-free and snow covered dataset, respectively, number of both fungal and bacterial ASVs in the network, i.e. associated to another ASV, total number of ASVs in the network, total number of associations in the network, longest path of the network and density of the network. Network properties were calculated using igraph (v1.2.6) [40]. In addition, the numbers of fungal-fungal, fb, and bacterial-bacterial associations and the numbers of associations detected in both snow-free and snow covered networks were compared within thresholds. All network characteristics were statistically tested across thresholds for differences among snow-free and snow covered soils using paired Kruskal test (Fig. 1). In order to study fb associations in snow-free and snow covered *P. cembra* soils, all inferred networks were subset to only fb associations.

In order to test if the frequencies of fungal-fungal, fb, bacterial-bacterial and shared associations between snow-free and snow covered networks observed in the inferred networks deviate from the frequencies expected by chance, we randomly generated 999 random networks for each network inferred (Fig. 1). More specifically, for the random networks, we used the ASVs present in the respective filtered dataset and from them, we randomly picked a number of random associations similar to the number of associations inferred from this respective filtered dataset. We repeated this process 999 times and for each random network, we determined the frequencies of fungal-fungal, fb, and bacterial-bacterial associations, thereby generating a random distribution as reference distribution for each network from the original data. In addition, within each combination of fungal and bacterial thresholds, we counted the number of total and fb associations 999 snow-free and snow covered networks shared by chance. The resulting distribution of randomly shared associations among snow-free and snow covered networks was compared to the number of shared associations actually observed in the matching snow-free and snow covered network pairs of each filter combination.

In the same manner as above, we randomly picked fb associations from those fungal and bacterial ASVs that were connected to a bacterial or fungal ASV in each respective network. In 999 randomly picked fb association sets matching each network inferred from the original data, we counted how many fb associations were shared between the respective snow-free and snow covered networks. Then, we counted the number of shared associations observed in both the original data and the random distribution. In comparison to the random associations picked in the above described procedure, considering fungal-fungal, fb, and bacterial-bacterial associations, in this random picking procedure, the number of shared associations by chance is higher due to the lower number of ASVs. This procedure, however, answers the question if fb associations are more likely to be kept or to be replaced by associations to different ASVs.

In order to answer the question if associations are more likely to be competitive or mutually beneficial, we calculated the edge weights for all associations included in each respective network by multiplying the symmetrized beta coefficients with their respective refit of the network structure. Please note that the edge weights calculated by SpiecEasi are based on penalized estimators and cannot be compared to correlation coefficients. We then calculated the odds of positive and negative associations in each network and modelled the influence of snow cover (snow-free vs. snow covered) on the odds ratio via generalized linear modelling assuming binomial distribution.

In order to answer the question if the association frequency differed among fungal phyla depending on snow cover, for each network, we grouped all fb associations by the phylum annotation of the fungal partner and counted how many times each fungal phylum associated to any bacterium. For each pair of snow-free and snow covered networks based on filtering criteria, we applied generalized linear modelling assuming Poisson distribution in order to estimate the association frequencies of all fungal phyla depending on snow cover. In other words, for each of the 25 filtering criteria separately, the association frequency of fungi to bacteria was modelled depending on the factors fungal phylum and snow cover.

For all generalized linear models (binomial and poisson), the best model was selected using stepwise forward and backward selection using R’s step command, which applyies Aikaike’s criterion.

Ggplot2 (v3.3.5) was used for part of the data visualization [41].

## Results

### Microbial communities of *Pinus cembra* forests are comparable and stable

**Fig. 2:**
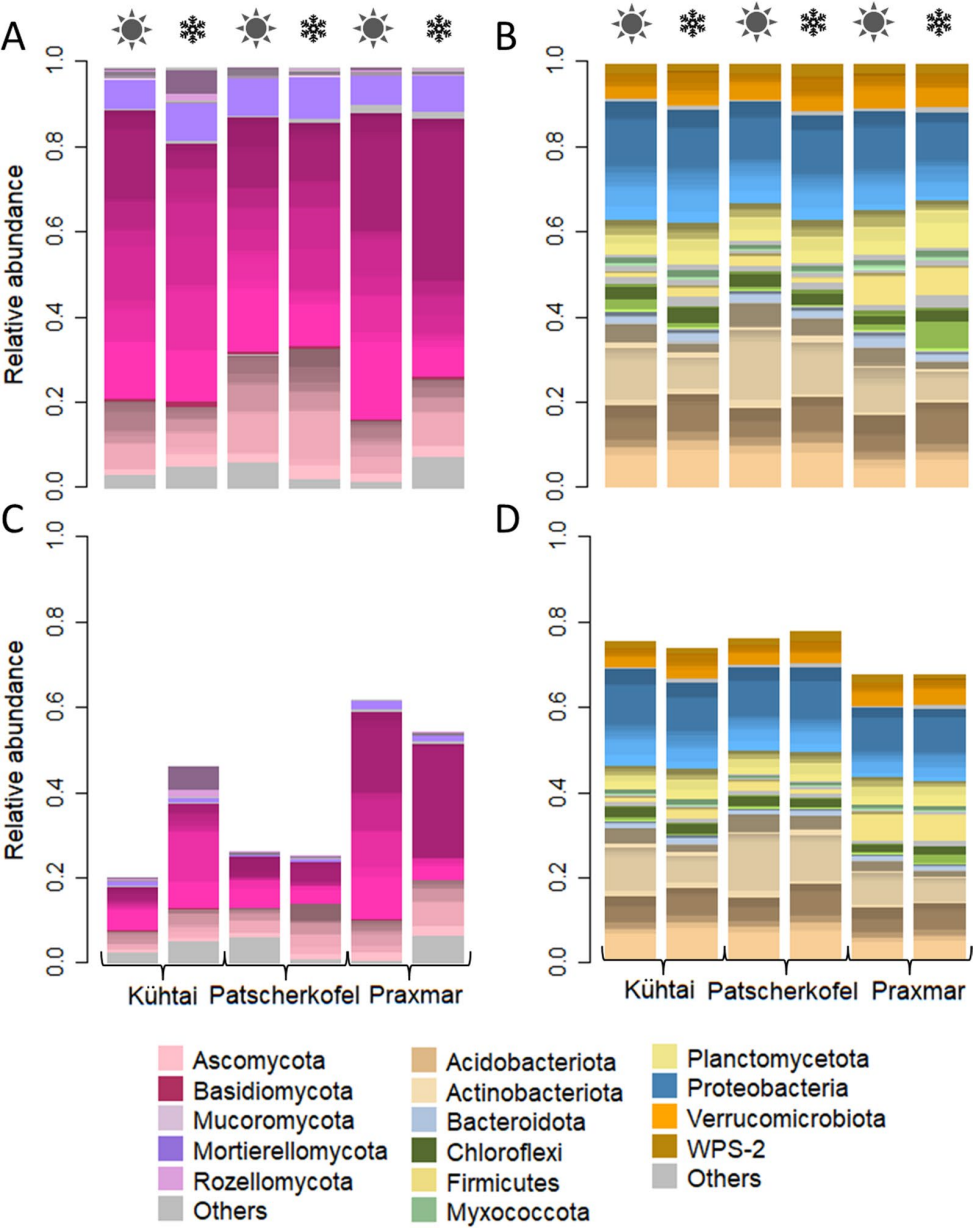
Overview of the microbial composition in all *Pinus cembra* forests investigated. **A**, **B** The taxonomic composition of the **A** fungal and **B** bacterial community at the different sampling sites is visualized separate for snow covered and snow-free soils. **C**, **D** The relative abundances of **C** fungal and **D** bacterial core ASVs (detected in at least one sample from all three locations) at the different sampling sites is visualized separate for snow covered and snow-free soils. Colour shades highlight the diversity of orders within phyla. The relative abundance was calculated as read proportion of microbial units referring to all reads within a sample group (ranging from 0 to 1, i.e. 0–100% of reads)

We hypothesized that (sub-)alpine *Pinus cembra* forests harbour a typical microbial community, i.e. microbes commonly detected in *P. cembra* forests, which might vary with season (snow cover). In order to test this hypothesis, we compared the fungal and bacterial community composition among sampling sites (Kühtai, Patscherkofel, Praxmar) and depending on snow cover. Based on the entire composition detected, the fungal community neither differed among locations (p_Adonis_ = 0.11, R^2^ = 0.029) nor between snow covered and snow-free soils (p_Adonis_ = 0.21, R^2^ = 0.014) (Fig. 2A, Additional file 2: Table S4). For bacteria, both location and snow cover had significant effects on the community composition, however, the variances explained by these factors were so low that differences were practically meaningless (R^2^_location_ = 0.036, R^2^_snow cover_ = 0.016, p = 0.001) (Fig. 2A, B; Additional file 2: Table S4). The comparability of the microbial communities among locations highlights that, in line with our hypothesis, our samples were well suited to represent and describe (sub-)alpine *P. cembra* habitats. Against our hypothesis, there was no evidence for a seasonal shift of the entire microbial community composition from snow-free to snow covered period or vice versa.

Further supporting our hypothesis of a comparable microbial community in different *P. cembra* forests, ASVs, which were detected at least once in all three locations, accounted for a high read abundance in all sample groups (Fig. 2C, D). These core ASVs we considered typical for *P. cembra* forests as they were detected independent of location. We will refer to them in particular throughout the manuscript. Both fungal and bacterial core ASVs were taxonomically diverse (Fig. 2C, D). For fungi, core ASVs accounted for on average 40%, 41%, and 27% of all reads in Kühtai, Patscherkofel, and at Praxmar, respectively. For bacteria, core ASVs accounted for an average of 75%, 77%, and 66% of all reads in Kühtai, Patscherkofel, and at Praxmar, respectively. While accounting for high percentages, the core ASVs were low in total numbers (93/1,654–5.6% fungal and 3,249/27,213–11.9% bacterial ASVs). It needs to be emphasized that ASVs, especially fungal ASVs, were usually detected in a small number of samples. On average, fungal core ASVs were detected in 12/79 samples (median = 8); bacterial core ASVs were detected in 14/79 samples (median = 9). This suggests a high spatial heterogeneity even within the same *P. cembra* forest.

Fungal richness (mean ± standard deviation = 45 ± 14 ASVs, *p*_Kruskal_ > 0.25) and Shannon diversity (2.6 ± 0.66, *p*_Kruskal_ > 0.15) were comparable across all locations (Additional file 1: Fig S4 A–C). Fungal diversity (Shannon) did not vary depending on snow cover (*p*_Kruskal_ > 0.09). However, fungal richness was higher in snow covered soils (50 ± 15 ASVs, n = 34) compared to snow-free soils (42 ± 13 ASVs, n = 45) (*p*_Kruskal_ < 0.012, Additional file 1: Fig. S5 A–C). Although seasonal differences were slim, this latter finding supports our hypothesis that *P. cembra* habitats at different locations were comparable, but varied depending on snow cover.

The bacterial alpha diversity, in contrast to the fungal one, differed among locations (*p* < 0.0045, Additional file 1: Fig. S4 D–F). Both bacterial richness and Shannon diversity were higher in Praxmar (1241 ± 268 ASVs, Shannon = 6.3 ± 0.36, n = 21) than at Patscherkofel (982 ± 290 ASVs, Shannon = 6.0 ± 0.35, n = 33) (p_Dunn.BonferoniHolm|richness_ = 0.0022, p_Dunn.BonferoniHolm|Shannon_ = 0.014). Bacterial alpha diversity in Kühtai (1053 ± 283 ASVs, Shannon = 6.2 ± 0.35, n = 25) was comparable to the ones in Praxmar and at Patscherkofel. These findings indicate that despite a high comparability of the overall bacterial community composition across locations, not all bacterial ASVs detected were typical for *P. cembra* forests and other factors might have influenced the bacterial community assembly. Snow cover did not affect the bacterial Shannon diversity (*p* > 0.3). The bacterial richness did not differ between snow covered (1143 ± 267 ASVs) and snow-free soils (1021 ± 312) (*p*_Kruskal_ = 0.058, Additional file 1:Fig. S5 D–F).

#### Typical *P. cembra* soil microbial communities

On the taxonomic levels of phylum and order, the compositions of the fungal and bacterial communities were generally comparable across locations and among snow covered and snow-free soils (Fig. 2). The fungal community was largely dominated by Basidiomycota with a comparably low percentage of Ascomycota reads. In the *P. cembra* forests studied here, there was also a large percentage of Mortierellomycotina (7–10% of the total reads) (Fig. 2A, C). The bacterial community was not dominated by one particular phylum; Proteobacteria, Acidobacteria and Actinobacteria were detected in comparable read percentages (Fig. 2B, D).

**Fig. 3:**
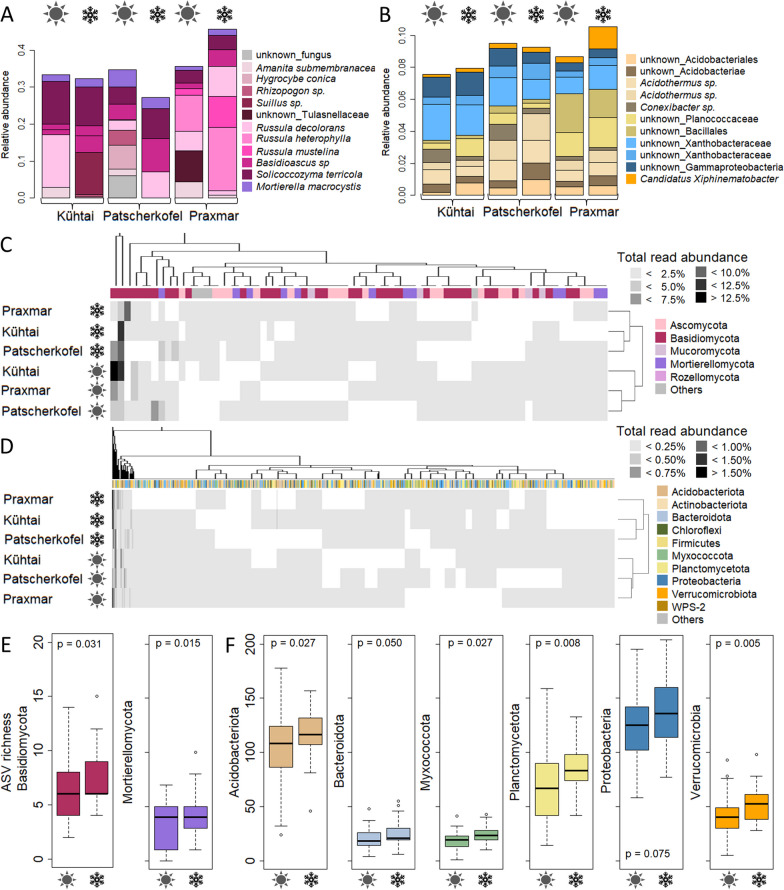
**A**, **B** Top abundant **A** fungal and **B** bacterial ASVs across all sample groups. Those ASVs with highest average relative read abundance within sample groups were selected. For fungi and bacteria, these ASVs accounted for > 7% and within 1.0–2.5% of the total read abundance, respectively. **C**, **D** Heatmaps of the core **C** fungal and **D** bacterial ASVs. **E**, **F** ASV richness within **E **fungal and **F **bacterial phyla. Only phyla with significant differences among snow covered and snow-free soils were illustrated; for bacteria, only phyla with ASV richness > 10 ASVs were displayed

For fungi, ASVs with high average abundances across samples usually accounted for > 7% (Fig. 3A). Six out of 14 fungal ASVs with high average abundances were core fungal ASVs. This implies that the abundance of the majority of core ASVs (93 ASVs) was lower than 7%. Two fungal high abundance ASVs annotated as *Solicococcozyma terricola* (7.0 ± 3.5% of all reads), a basidiomycete yeast, and *Mortierella macrocystis* (2.4 ± 1.2% of all reads), a saprobic soil fungus, were frequently detected in high relative abundances across all locations and independent of snow cover (Fig. 3A). In addition, two ASVs, both annotated as *Russula decolorans*, an ectomycorrhizal basidiomycete, were highly abundant in few (1–3) samples at each location, reaching up to 70% of all reads. Furthermore, one ASV annotated as *Amanita submembranacea* and one ASV annotated as *Hygrocybe conica* were detected across all locations. These two latter are agaricoid, potentially ectomycorrhizal basidiomycetes. While *Basidioascus* ASVs were not consistently detected across all locations, two different ASVs of this genus, which were not further annotated, appeared frequent and highly abundant in all *P. cembra* forests (Fig. 3A). This confirms that this genus typically occurs in forest habitats [42], but the species distribution depended on the location.

*Rhizopogon* species are hypogeneous ectomycorrhizal fungi commonly detected in *Pinus* forests [9, 11, 43]. Here, one unknown *Rhizopogon* ASV was detected in all locations. Three other *Rhizopogon* ASVs were found in the dataset, thereof one, also not identified on species level, with very high abundance. None of the *Rhizopogon* ASVs’ occurrences followed a seasonal pattern.

Interestingly, core ASVs did not contain *Suillus,* which is often reported as an ectomycorrhizal specialist in association with *Pinus* [9, 11, 43]. Here, there were six ASVs annotated as *Suillus*; four of them were annotated as *S. plorans*, the other two did not have species annotation. Two of the *S. plorans* ASVs were detected four and seven times and independent of snow cover, the other *Suillus* ASVs were detected once.

For bacteria, high average ASVs abundances were 1–2.5% of all reads. Usually, these bacterial ASVs of high abundances were not annotated on a fine taxonomic level (Fig. 3B). However, they were taxonomically diverse, belonging to the phyla Proteobacteria, Acidobacteria, Actinobacteria, Firmicutes and Verrucomicrobia.

#### Seasonality—fungal richness is higher in snow covered soil and the composition partly changes

Although there were no (relevant) differences in *P. cembra* soil microbial compositions or richness according to season, neither in the overall dataset nor among core ASVs (Fig. 2; Additional file 1:Fig. S4, S5; Additional file 2: Table S4), we found some fungal and bacterial ASVs that were detected across all locations, but exclusively detected in either snow covered or snow-free soil samples (Fig. 3C, D). Furthermore, some fungal and bacterial phyla were richer/poorer in their numbers of ASVs detected during snow covered and snow-free periods (Fig. 3E).

The abundance and occurrence of one of the highly abundant *Russula decolorans* ASVs that was identified as typical for *P. cembra* forest soils (core ASV) appeared influenced by snow cover: In Kühtai, this ASV was detected only under snow-free conditions. In Praxmar, its abundance reached high percentages (40% of all reads, 3 samples) in snow-free conditions only (compared to percentages below 10% in the 2 snow covered samples). However, at Patscherkofel, the abundance of this ASV was independent of snow cover. In addition, another Basidiomycota ASV annotated to the genus *Trechispora* was solely detected in snow-free soil samples (6/45 samples) and a fungal core ASV annotated as *Tetracladium marchalianum* was found only in snow-free samples. Exclusively under snow covered conditions, a Basidiomycota core ASV annotated as *Phenoliferia psychrophenolica* was detected (9/35 samples). Additional core ASVs, detected exclusively in snow covered and snow-free conditions, were found, although in a lower number of samples (< 6 samples) (Fig. 3C) (Additional file 2: Table S5).

In addition to these compositional differences, we detected a higher richness of Basidiomycetes in snow covered compared to snow-free soil (Fig. 3, Additional file 2: Table S6). Similarly, Mortierellomycotina were higher in richness in snow covered compared to snow-free soil.

Bacteria were more evenly distributed than fungi. There were higher numbers of bacterial than fungal core ASVs detected in both snow covered and snow-free soils (Fig. 3D). This is in line with their much higher richness compared to the fungi. However, the taxonomic composition of those core ASVs that were abundant across samples differed depending on snow cover: Core ASVs exclusively detected in snow covered soils and frequently detected across samples (> 5 samples) were frequently annotated to Proteobacteria (5/11 ASVs) and Verrucomicrobia (2/11 ASVs). Core ASVs exclusively detected in snow-free samples belonged to the phyla Actinobacteria (2/8 ASVs) and Myxococcota (2/8 ASVs), respectively. On a finer taxonomic level, no commonalities were found (Additional file 2: Table S7).

The richness within bacterial phyla in snow-free soil was never higher compared to the one in snow covered soils. The richness of the phyla Acidobacteria, Bacteroidota, Myxococcota, Planctomycetota and Verrucomicrobia was higher in snow covered than in snow-free soils (Fig. 3, Additional file 2: Table S8).

### Fungal-bacterial associations were more frequent and diverse in snow covered than in snow-free soils

**Fig. 4:**
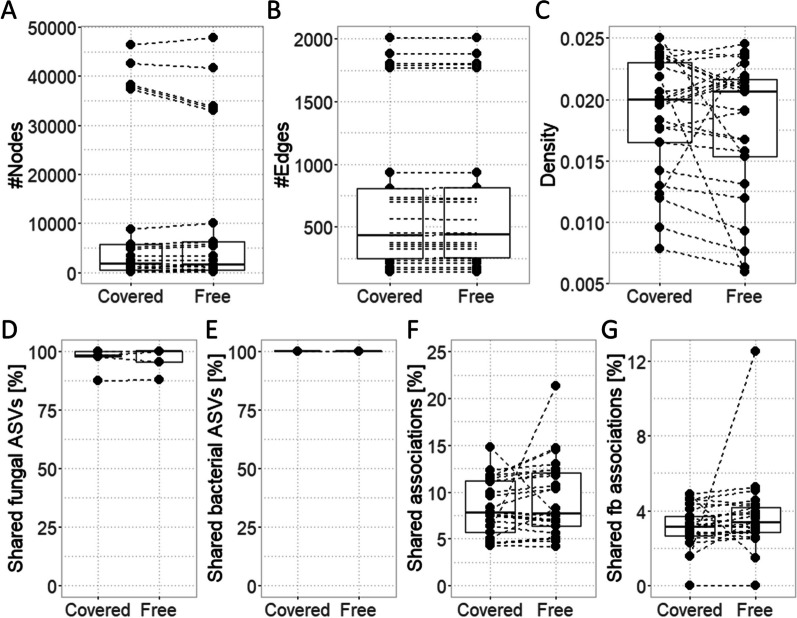
Comparison of snow-free and snow covered association networks. Points connected by dashed lines refer to network pairs, i.e. snow-free and snow covered networks inferred after applying different filtering criteria. **A** The number of nodes in the networks. **B** The number of edges = associations in the networks. **C** The density of the networks as the number of inferred associations relative to the number of possible associations. **D**, **E** The percentages of **D** fungal and **E** bacterial ASVs detected in both the snow-free and snow covered network of each network pair. **F**, **G** The percentage of associations detected also in the paired network: **F** all associations considered (fungal-fungal, fungal-bacterial, and bacterial-bacterial associations), **G** only fungal-bacterial (fb) associations considered. Boxes depict the first to third quartile, whiskers indicate the maximum value to the third quartile, the black line indicates the median

Association networks inferred from neither snow-free nor snow covered soils were dense (Fig. 4A-C) and they did not differ in density (*p*_Wilcox_ = 0.21): From all possible edges, a median of 1.8% edges were realized in both networks (the maximum was 2.5% edges realized). Neither the number of ASVs nor the numbers of connections among ASVs differed in an ecologically meaningful manner between networks (Fig. 4B, C; Additional file 2: Table S9). In the snow-free network, the percentage of bacterial ASVs connected into the network was slightly lower compared to the snow covered network (*p*_Wilcox_ = 0.0318): While in > 50% of the networks calculated, all bacterial ASVs were integrated into the network, the lowest snow-free quartile contained only 75% of bacterial ASVs, whereas the snow covered network contained 98% of all bacterial ASVs. With only few exceptions, all fungal ASVs were integrated into both networks; there was no difference in this regard comparing the snow-free and the snow covered network.

Despite a high percentage of ASVs used for inferring both the snow-free and snow covered network, only a relatively low percentage of associations were detected in both snow-free and snow covered networks (Fig. 4D–F). Underlining the ecological relevance of the inferred associations, the numbers of associations observed in both networks was higher than expected from random picking (Additional file 1: Fig. S6, Additional file 2: Table S10). In both, snow-free and snow covered networks, bacterial-bacterial associations accounted for the majority of associations (1st–3rd quartile = 67–89%; Additional file 1: Fig. S7). Compared to associations drawn randomly from the fungal and bacterial ASVs subjected to network inference, the observed number of bacterial-bacterial associations exceeded the random expectation in both the snow-free and snow covered network (Additional file 1: Fig. S6). Fungal-bacterial associations were the second most frequent associations detected in both inferred networks (8–14%; Additional file 1: Fig. S7). As for bacterial-bacterial associations, the percentage of fb associations observed in the inferred snow-free and snow covered networks was higher than expected from the overall random associations (Additional file 1: Fig. S6). Only few fb associations were detected in both snow-free and snow covered networks (Fig. 4G), which was in line with the expectations from random pickings (Additional file 1: Fig. S6, Additional file 2: Table S10). The percentage of fungal-fungal associations was lowest (1–14%; Additional file 1: Fig.S 7). In both the snow-free and snow covered network, fungal-fungal associations were less frequent than expected from random picking (Additional file 1: Fig. S6).

Considering only fb associations, the snow covered network was denser compared to the snow-free network (*p* = 0.0026), although the difference in density was small (median_snow-free_ = 0.0297, median_snow covered_ = 0.0315). While in both networks the majority of associations were positive, the odds of positive association were lower in snow covered (0.60) compared to snow-free networks (0.64) as analysed using binomial model (*p* <  < 0.01; Additional file 2: Table S11). It should be noted though, that based on the networks, we do not want to infer symbiosis or mutual exclusion as for such conclusion, the edge weights between nodes were too low (Additional file 1: Fig. S8).

**Fig. 5:**
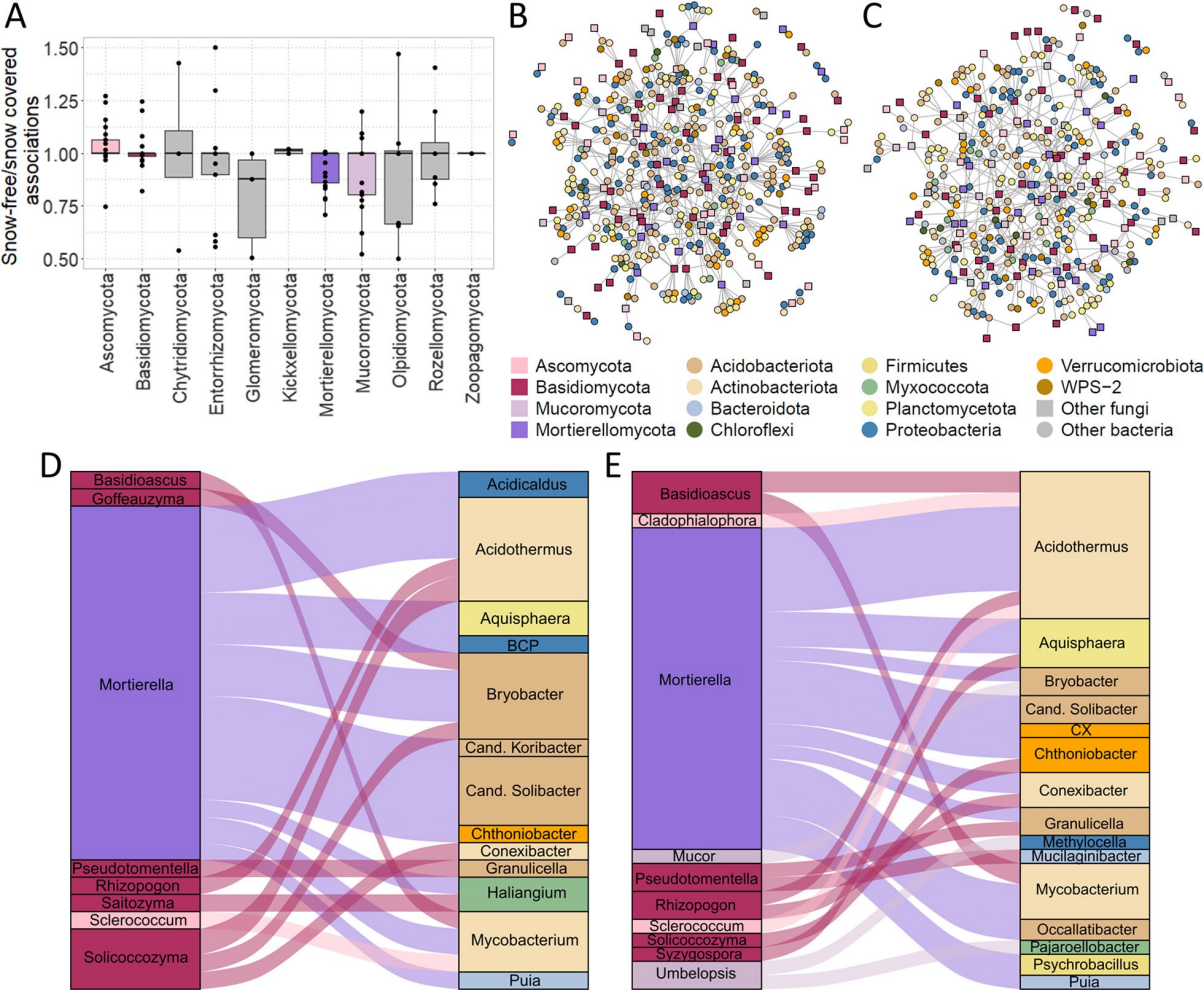
Fungal-bacterial (fb) associations frequently detected in snow-free and snow covered networks. **A** The influence of snow cover on the fb association frequency of fungal phyla was modelled using generalized linear model assuming Poisson distribution. Each point represents the estimate predicted by generalized linear modelling assuming Poisson distribution. **B**, **C** Frequent fb associations in **B** snow-free and **C** snow covered networks. For panels B–E, associations observed < 5 times across networks were omitted from display. **D**, **E** The Sankey plots visualize frequent fb associations summarized on genus level. Here, only those frequent fb genera associations are displayed that (i) received a taxonomic annotation on genus level and (ii) that were observed at least twice. The total number of associations displayed in the **D** snow-free and the **E** snow covered network (bars) is 20 and 25, respectively. Cand. = Candidatus, BCP = *Burkholderia-Caballeronia-Paraburkholderia*, CX = Cand. *Xiphinematobacter*

In order to go into more detail on specific fb associations, we first fit Poisson models to the networks estimating the frequencies with which a fungal phylum was associated to bacteria, if significant, depending on season (Fig. 5A, Additional file 2: Table S12). While the main effect of snow cover was usually insignificant, for the abundant fungal phyla, their association frequency often depended on snow cover: While Ascomycota and Basidiomycota ASVs associated to bacteria more frequently in snow-free than in snow covered networks, Mortierellomycotina and Mucoromycotina ASVs associated to bacteria more frequently in snow covered than in snow-free networks (Fig. 5A; Additional file 2: Table S12). Next, we searched for and compared fb associations detected frequently (> 5 networks) across snow-free and snow covered networks. A high number of associations occurred in only one or few networks; others were frequently detected (Additional file 1: Fig. S9). In all networks, fungal phyla were usually associated to different bacterial phyla, indicating a high diversity of fb associations (Fig. 5B, C).

In line with our hypothesis that there would be a small set of seasonally independent associations characteristic for *P. cembra* forests, the majority of frequent associations was detected either in networks inferred from snow-free or snow covered soils, but not in both (Additional file 1: Fig. S9). This set involved core ASV associations annotated to several fungal and bacterial phyla (Additional file 2:Table S13). Frequent associations (> 5 networks) detected in both snow-free and snow covered networks were often among Basidiomycota ASVs, mainly *Basidioascus* (6 frequent associations) and *Solicoccozyma* (2 frequent associations), and ASVs belonging to different bacterial phyla. Frequent associations involving Ascomycota were detected between ASVs annotated as *Cladophialophora* and *Acidothermus* (Actinobacteriota), *Sclerococcum* and *Acidibacter* (Proteobacteria), *Bryssonectria* and *Psychrobacillus* (Firmicutes), and *Nadsonia* and a not further annotated Elusimicrobiota ASV. Three different *Mortierella* ASVs were associated to *Mycobacterium* (Actinobacteriota), *Bryobacter* (Acidobacteriota)*,* and an unknown Acidobacterium ASV. An *Umbelopsis* ASV (Mucoromycota) was associated to a bacterial ASV annotated as *Acidicapsa* (Acidobacteriota).

In line with our hypothesis that fb associations depend on snow cover, the majority of frequent fb associations was detected either in snow-free or in snow covered networks (> 85%, Fig. 4G). For investigating these frequent fb associations (> 5 networks) more closely, we summarized them on genus level (Fig. 5D, E). In the following presentation of frequent fb associations in snow-free and snow covered *P. cembra* forests, those fungal taxa known to be relevant for the ecosystem will be focussed.

In snow-free and snow covered conditions, the ectomycorrhizal *Basidioascus* was associated to Mycobacterium (Fig. 5D, E). In addition, under snow covered conditions, four *Basidioascus* ASVs were positively associated to *Acidothermus* ASVs. In snow-free conditions, associations were weaker and positive. Ectomycorrhizal *Pseudotomentella* was negatively associated to *Granulicella* independent of snow cover (Fig. 5D, E). Under snow cover, *Pseudotomentella* was also mostly negatively associated to *Mucilaginibacter*. This association was not among the frequent associations in snow-free conditions, thereby making it specific for snow covered conditions. Ectomycorrhizal *Rhizopogon* was negatively associated to *Acidothermus* and *Conexibacter* (Fig. 5D, E). Snow cover did not seem to affect these associations, as there were two frequent associations between *Rhizopogon* and *Acidothermus* in each snow-free and snow covered soils and one/two frequent associations between *Rhizopogon* and *Conexibacter* in snow-free/-covered soils. Similarly, though positively, *Cladophialophora,* was associated to *Acidothermus* (Fig. 5D, E). *Cladophialophora* ASVs were usually annotated as *C. sylvestris,* which is non-pathogenic and saprobic.

Frequent associations among Basidiomycota and Ascomycota and bacteria were often independent of snow cover. However, saprobic Mucoromycotina associated to bacteria predominantly under snow cover (Fig. 5D, E): a mainly positive association among *Mucor* and *Bryobacter*, which is an acidotolerant, slow-growing, chemoorgantrophic soil bacterium [44]; a negative association among *Umbelopsis* and *Methylocella*, which is facultative aerobic methanotrophic bacterium able to utilize ethane, propane, and alkanes [45]; and a mainly negative association among *Umbelopsis* and the monotypic *Pajaroellobacter* (*P. abortibovis*), which was associated with bovine abortion [46].

The main share of frequent fb associations in both snow-free and snow covered networks was detected for *Mortierella* (Fig. 5D, E). There were mainly negative associations among *Mortierella* and *Aquisphaera,* and both, positive and negative associations among *Mortierella* and *Mycobacterium.* ASV numbers involved in these associations were almost twice as high in snow covered as in snow-free soils. In addition, *Mortierella* associated frequently with *Acidothermus,* Cand. *Solibacter,* and *Bryobacter.* The associations to Cand. *Solibacter* were mostly negative under snow cover and positive under snow-free conditions (Fig. 5D, E).

*Solicoccozyma*, which was a core fungus here and is typically found in *P. cembra* forests, was found positively associated to *Acidothermus* under both snow-free and snow covered conditions (Fig. 5D, E). The associations involving *Acidothermus* and *Bryobacter* were made up by two frequent ASV associations under snow-free conditions; one frequent ASV association was detected under snow covered conditions. In addition, *Solicoccozyma* associated mainly negatively with *Bryobacter.* Associations among *Solicoccozyma* and *Conexibacter* were positive under snow cover, but mainly negative under snow-free conditions.

There were few frequent associations among *Russula* and *Aquispheara, Conexibacter,* and *Methylocella,* respectively, under snow covered conditions. While associations to *Aquisphaera* were positive, the associations to *Conexibacter* and *Methylocella* were mainly negative. Under snow-free conditions, one frequent positive association of *Russula* and *Bryobacter* was found.

## Discussion

### Microbial communities typical for *P. cembra* forests and how they depend on snow cover

Overall, the microbial community composition did not differ among locations and depending on snow cover. With regards to location, all forests studied here were independent *P. cembra* habitats from similar climatic regions and with similar soil pH. As these factors are the major drivers of compositional differences in soil fungal and bacterial communities [47, 48], these results are in line with our hypothesis and the literature. Interestingly, our results contrast a previous study focussing on actual ectomycorrhizal associations of *P. cembra* from comparable locations in South Tyrol (Italy) [11]. In this study, location accounted for about 17% of the variation observed in the *P. cembra* root ectomycorrhizal compositions. One explanation for this difference might be that here, bulk soil was studied, while [11] focussed specifically on root-colonization. Bulk soil communities represent the ectomycorrhizal potential of a habitat. The actual mycorrhization of the plant root might be regulated by the host plant, depending on its specific needs and community dynamics during the establishment of mycorrhizal associations. As the probability of detecting temporal and seasonal differences in communities decreases with increasing sequencing depth [49], the contrasting results between these two studies, involving soil high throughput sequencing and clone libraries of mycorrhized root tips, respectively, might also be purely methodological. Another reason might be that the Austrian and South Tyrolean *P. cembra* stands differ in climatic conditions.

Spatial local-scale heterogeneity was high, as typical for mountain soils [2, 11, 19, 50]. Many partially highly abundant ASVs were distributed heterogeneously, and bacterial and especially fungal ASVs were usually detected in only very few samples. The high spatial heterogeneity enforced defining a quite low threshold forASVs typical for *P. cembra* forests (core ASVs, i.e. ASVs detected at least once in every forest location). In low heterogeneity habitats, the core is often defined by microbial units detected in around 50% of the samples within each sample group. Given the high heterogeneity in (sub-)alpine forests, a core set of microbes available in all or almost every *P. cembra* sample might not exist, thereby underpinning the high diversity and versatility of this ecosystem. Therefore, and due to the fact that the core detected was relatively small (5.6% and 11.9% of the total fungal and bacterial ASVs detected), we think it is reasonable to assume that the core ASVs detected partially cover the versatility of (sub-)alpine *P. cembra* forests and can be considered typical for this habitat. Supporting the assumption of their relevance, core ASVs accounted for high abundances in the samples they were detected in, in sample groups and in the overall dataset. In line with the versatility of the fungal composition present in *P. cembra* forests, the fungal richness and diversity were comparable among locations, as observed previously in other locations [11]. The bacterial diversity, however, differed among locations, thereby indicating that the distribution of bacteria across *P. cembra* habitats might be influenced by other factors than the fungal one, e.g. dispersal limitation [51].

Here, the fungal and bacterial community composition in snow-free and snow covered *P. cembra* forest soils was similar, thus contradicting our hypothesis of a snow cover effect. A significant effect of snow cover was reported for both fungal and bacterial communities in a mixed boreal forest consisting of *Pinus sylvestris* and *P. abies* [19]. Similarly, the microbial transcriptomes of a *P. abies* forest were reported to differ depending on snow cover [18, 54]. Interestingly, on the level of DNA, these authors did not find compositional differences between seasons, which is in line with our study. On RNA level, the relative contribution of saprotrophs to the overall transcription was higher in winter than in summer, while the contribution of ectomycorrhizal transcripts was higher in summer compared to winter [54]. Such functional shifts in fungal community composition were also reported from a northern pine forest, where saprotrophs dominated under the snow and ectomycorrhizal fungi dominated during the vegetation period [17]. Such shift in dominance was not detected in our study, which might be due to altitudinal effects. Another reason for the high similarity of the microbial community composition in snow-free and snow covered soils, might be a masking effect of extracellular DNA, which can persist in ecosystems for a considerable while [55, 56]. In line with our hypothesis on seasonal dynamics of soil microbial communities in (sub-)alpine *P. cembra* forests, we found a higher richness of fungi in general, and of several fungal and bacterial phyla in snow covered compared to snow-free soils (Fig. 3E, F). Particularly, we detected a higher richness of Mortierellomycotina, Basidiomycota, Bacteroidetes and Proteobacteria in snow covered compared to snow-free soils (Fig. 3E, F). Mortierellomycotina are well known for their ability of degrading organic matter [59]. Basidiomycota are known for their role in C-cycling as they can degrade (dead)wood, including lignin [60, 61]. Similarly, copiotrophic Bacteroidetes, as well as copiotrophic Proteobacteria are involved in litter degradation [62]. A higher richness of taxa involved in the degradation of complex C-rich polymers indicates that litter and soil organic matter degradation continues under snow cover similarly to snow-free conditions, but potentially by (a higher number of) different organisms. The continuous litter input in evergreen forests and the slow decomposition of recalcitrant needles might foster the comparability of the microbial community across seasons by promoting similarities in microbial functionality. López-Mondéjar and colleagues [16] studied litter and soil bacterial communities in *Quercus* forests under snow-free and snow covered conditions, and found compositional differences among both, soil layers (soil and litter) and seasons, with the highest seasonal impact on the litter layer. In this regard, it would be interesting to study the transcriptomes of *P. cembra* forests (studied here) in separate soil layers, in order to shed light into the seasonal dynamics of soil organic matter decomposition in coniferous forests under snow cover.

Several typical and relevant taxa were clearly depending on snow cover (Fig. 3A-D; Additional file 2: Table S5). Some species’ ASVs, especially *Russula,* were most abundant only under either snow-free or snow covered conditions (Fig. 3A) indicating a seasonally depending competitive fitness. *Russula* is a typical ectomycorrhizal basidiomycete of *P. cembra* forests [9, 11]. However, it is likely that *Russula* species also have good saprobial capacities, thereby allowing their growth under snow cover.

*Suillus (plorans)* ASVs were frequently detected, although none of these ASVs was detected across all locations (no core ASVs). *Suillus plorans,* also known as Swiss pine bolete [63], is an ectomycorrhizal fungus specific for *P. cembra* trees. The reason why, here, *Suillus* was not detected as member of the core microbiome might be methodological: *Suillus* spp. form extensive networks consisting mainly of thick rhizomorphs, which only re-differentiate to form adsorptive hyphae when needed (e.g. on rocks). Moreover, there is a high intra-species ITS sequence variability in *Suillus* species (in contrast to *Russula* spp. and *Cortinarius* spp. with a very low divergence). Here, several ASVs were assigned as *S. plorans.* Overall, the species has a high occupancy across all samples. We deduce that these factors have masked the wide distribution of *Suillus* species, including *S. plorans* in our *P. cembra* habitats.

The genera *Trechispora* and *Tetracladium* (core ASVs) were detected solely in snow-free soil. *Trechispora* is a saprobic and potentially ectomycorrhizal Basidiomycete [66]. *Tetracladium* has a dual life cycle occurring as plant endophyte in terrestrial habitats, and as saprobial aquatic fungus [67, 68]. Their occurrence during the vegetation period is in line with plant-associated, mutualistic fungi being more active in summer than in winter [17, 54].

Here, *Phenoliferia psychrophenolica* was a core species detected exclusively under snow cover. This basidiomycete yeast is typical for cold habitats [71, 72] and it cannot grow at temperatures higher than 15 °C [73]. As it can degrade phenolic compounds, it might contribute to the degradation of litter under snow cover in alpine habitats. Therefore, it might a key species for nutrient turnover in (sub-)alpine snow covered *P. cembra* forest soils.

### Fungal-bacterial associations are influenced by season

This is the first study approaching fb associations in (sub-)alpine *P. cembra* forest considering seasonal differences (snow cover), a factor which is tremendously understudied [74]. Our data provide a solid base for understanding microbial associations and their dynamics, which is fundamental for a better understanding of multipartite interactions like ectomycorrhizal mutualism.

In line with our hypothesis that snow cover influences fb associations, networks inferred from snow covered soils were denser than those from snow-free soils (*p* = 0.0026; median_snow-free_ = 0.0297, median_snow covered_ = 0.0315) and they integrated slightly more bacterial ASVs. Moreover, we detected slightly higher odds of positive fb association in snow-free (0.64) compared to snow covered soils (0.60; *p* <  < 0.01). This supports differences in nutrient access and utilisation depending on snow cover [13]. For *P. abies* forests, a seasonal partitioning of work among fungi, which degrade complex carbohydrates in summer, and bacteria, which degrade storage carbohydrates in winter, has been suggested [54]. This does not contrast the relevance of fb associations, whose relevance is supported by the higher number of inferred fb associations compared to the number expected by chance for both snow-free and snow covered soils. Likely, the seasonal changes in nutrient cycles [4, 54, 75, 76] are associated with differences in fb associations. It is likely that soil microbes adopt a competitive and stress tolerant strategy when using resources under snow cover, whereas they adopt a more cooperative strategy during snow-free periods.

Independent of snow cover, fungal ASVs had a higher connectivity than bacterial ASVs (Fig. 5B, C). This is not surprising, given that litter in coniferous forests represents a complex nutrient source that is not easily degraded and that fungi are considered the primary decomposers of such complex substrates [77]. Usually, fungal phyla were associated to bacterial ASVs annotated to different bacterial phyla (Fig. 5), indicating a high diversity of fb associations. This emphasises the ecological association among very different organisms, thereby also emphasizing that complex ecosystem cannot be represented or researched fully by studying individual, selected taxa. While Ascomycota and Basidiomycota ASVs associated to bacterial ASVs more frequently in snow-free than in snow covered soils, Mortierellomycotina and Mucoromycotina ASVs associated to bacteria more frequently in snow covered than in snow-free soils (Fig. 5A). The dominance of Ascomycota in snow-free conditions might be due to their wide distribution and high species richness [78]. Many ectomycorrhizal fungi are Basidiomycota, which particularly colonize the roots of their host plants during the growing season [12]. Saprobic Mortierellomycotina accounted for a high abundance throughout the year, which is likely owed to their high abundance in (sub-)alpine ecosystems [79, 80]. They can grow at low temperatures [79], which explains their high association frequency under snow cover. Their higher association frequency under snow covered compared to snow-free soil suggests that bacteria might contribute to the competitive advantage Mortierellomycotina appear to have under these conditions.

One frequent positive fb associations observed was *Cladophialophora* (Ascomycota) and *Acidothermus* (Actinobacteriota). There was no indication of snow cover effect on this association. *Cladophialophora* spp*.* are wide-spread “black fungi”, which on the one hand have been described as soil saprobes or parasites to lichens and on the other hand can be pathogenic to humans and plants [81]. *Acidothermus cellulolyticus* is the only species described in the family of *Acidothermaceae* [82]. It is a thermophilic and acidophilic organism known to produce thermostable cellulose degrading enzymes [82]. ASVs annotated as *Acidothermus* (without species annotation) have frequently been detected in this study (287 ASVs, Fig. 3B) and they were frequently associated to different fungal ASVs, e.g. *Rhizopogon, Solicoccozyma, Basidioascus* and *Mortierella* (Fig. 5D, E). One can argue that this ASV was wrongly annotated or that the corresponding organisms might be inactive. We prefer to argue that there might be undiscovered *Acidothermus* species in (sub-)alpine soils, especially as this genus has been detected in both agricultural and reclaimed soils [83, 84]. These *Acidothermus* species might also have cellulolytic capabilities and they may play a role in degradation of complex polymers in coniferous forest soils. However, further studies are necessary to investigate both the identity of the ASV corresponding organisms and their frequent association to different fungi from different phyla.

Here and elsewhere [11], the ectomycorrhizal genus *Russula,* mainly *R. decolorans,* was an abundant *P. cembra* core species. *Russula* species diversity is probably high, as we found different *Russula* ASVs. Abundant core *Russula* ASVs associated with different bacteria depending on snow cover. Under snow cover, they positively associated to *Aquisphaera* (Planctomycetota).*.* The two known aquatic species of *Aquisphaera* have an incomplete nitrification pathway, thereby lacking the gene coding for ammonia monooxygenase [85]. *Russula* has acquired an evolutionarily conserved genetic profile for N acquisition and it specializes in ammonium rather than NO_2_ uptake [86]. Thus, the association among *Russula* and *Aquisphera* might evolve around N-utilization, especially when considering that snow cover influences N-cycling [13]. Under snow-free conditions, *Russula* positively associated with *Bryobacter* (Acidobacteriota). This genus accommodates one acidotolerant, strictly aerobic, slow-growing chemoorganotrophic species, *B. aggregatus*, which inhabits acidic wetlands and soils and appears to cohabit with *Sphagnum* moss [90]. In sesame cropping, *Bryobacter* was found positively related with plant health [91]. This indicates that it might play a role in the ectomycorrhizal functioning of its fungal partner.

The ectomycorrhizal basidiomycete *Pseudotomentella* negatively associated to *Granulicella* (Acidobacteriota) independent of season (Fig. 5D, E). Known *Granulicella* can hydrolyse plant polymers, e.g. xylane and pectane [92]. This could explain the plant-protecting properties as attributed to many ectomycorrhizal fungi acting as antagonists to plant pathogens. Under snow cover, *Pseudotomentella* was also associated to *Mucilaginibacter* (Bacteroidota) (Fig. 5D, E), which is an endophytic plant growth promoting bacterium able to hydrolyse xylan and pectin [93, 94]. It could also serve as mycorrhizal helper bacterium, as especially under high salt concentrations, plants might benefit from its presence [94]. We speculate that such fb associations might maintain ionic balances in the rhizosphere at zero temperatures.

Here, several ASVs were annotated as *Mortierella,* mainly *M. macrocystis,* and they were frequently and abundantly detected across samples and seasons (Figs. 2A, 3A, C). This is in line with [17] who consistently detected *Mortierella* among the top ten abundant genera across soil layers and time. For this genus, fb interactions were extensively studied in vitro, thereby allowing us to compare in vitro data with our in vivo assessment of associations: Selected *Mortierella* spp. are often associated to endohyphal bacteria, such as *Mycoavidus*, *Mycoplasma*-related or *Burkholderia*-related bacteria, or to epihyphal bacteria, such as *Pseudomonas* spp. [80, 96]. Here, we did not detect *Mortierella* associations with any of these endo- or epihyphal bacteria. However, above-mentioned reports originate from fungal pure culture isolates associated to bacteria, not from in vivo assessments. The fact that previously reported in vitro associations were not detected might therefore have methodological reasons (see below). It might also be due to low frequency of association or heterogeneous occurrence of the associations reported, which then cannot be detected in the networks. Nevertheless, we found *Mortierella –* bacteria associations (Fig. 5D, E): Independent of snow cover, *Mortierella* was negatively associated to *Aquisphaera.* Like *Russula*, under snow-free conditions, *Mortierella* associated with *Bryobacter* (Fig. 5D, E). Under snow cover, *Mortierella* also associated with Candidatus *Solibacter.* Under snow-free conditions, the association was positive, while it was negative under snow cover. Both, *Bryobacter* and Cand. *Solibacter* have been associated with plant health and growth promotion [91]. Interestingly, also *Mortierella* spp. are considered as plant beneficial [97], and these associations might underscore the plant-growth enhancing potential of these fungi. The seasonally dependent directionality of the *Mortierella –* Cand. *Solibacter* associations indicates a potential role of the plant in this association, thereby making it a good candidate to study tri- or multipartite interactions. In (sub-)alpine ecosystems, *Mortierella* is an important part of the core microbiome, which is active under snow cover [47, 79]. It is very likely that *Mortierella* and associated bacteria synergistically colonize soil habitats, and that metabolic products, like fatty acids produced by *Mortierella,* serve as substrates for associated bacteria.

The differences in associations among fungal and bacterial phyla and genera support seasonal changes in microbial ecology depending on snow cover, which is in line with our hypotheses. Different taxonomical and functional groups like ectomycorrhizal basidiomycetes (see above) and multifunctional Mortierellomycotina and Mucoromycotina were associated to a wide range of bacterial phyla. This highlights the need to improve our understanding of microbial interactions.

In soil, a large number of different microbes and microbial populations coexist in a finite space. Soil is heterogeneously colonized by microbes, and for microbes, soil aggregates may or may not be connected. Consequently, microbes may or may not be in close physical contact or interacting with each other in some way. This challenges the classical, rather narrow concept of species interaction. We think it is possible that interaction in a wider sense might better be understood as interdependence forming an intricate, but complex net. This is all the more compelling when we consider that all microbial interactions ultimately evolve around nutrients, which might be exchanged in close physical contact or over longer distances. As the phenomenon of mycorrhization shows, the microbiome cannot be clearly separated from the plant. This calls for studies that combine mechanistic direct interaction experiments and ecosystem level research.

### Methodological aspects

Here, we inferred networks across different filtering criteria and we interpreted only those associations that were frequently detected across criteria, i.e. networks, and whose taxa involved have literature support. This approach has not been applied elsewhere and network inference has a mixed reputation. Therefore, we will critically discuss this approach. This is particularly relevant, as interpreting associations across filtering criteria can be easily transferred to other studies. Therefore, we will address six frequently criticized points and argue how our experimental design and data analytical approach can help resolve these issues.

1. Interpretation of inferred associations as close or intimate interactions. We hypothesized that the network structure and microbial associations would depend on snow cover. We did not make claims on actual interactions. Our data support this conclusion independent of whether or not microbes interact, associate or co-occur.
2. Sampling size and robust inference [24, 100]. In order to have an as high sampling size for network inference as possible, we did not use networks of different locations for studying the effect of snow cover on the network and associations (n = 3, with low number of samples per network), but calculated the networks across locations and compared associations across filtering criteria (n = 50, with high number of samples per network). This was possible without biasing the results due to the comparability of the locations. One can argue that these replications are pseudo replications. However, they are necessary to evaluate the network inference, accounting for its technical limitations. Under the filtering conditions applied here, the different criteria applied provide ecologically meaningful networks and allow for their comparison. Replicate differences between representative snow-free and snow covered networks remain highly likely ecological differences, even though inference performance cannot be evaluated as true associations remain unknown. In addition, even if networks were calculated for several locations, the influence of filtering criteria on the associations predicted remains unknown and might bias the results to an unknown extent.
3. Indirect associations, i.e. cases of two microbial units associate via a third one, and cross kingdom associations, which require proper combination of two compositional datasets prior to network inference, and both compositionality and sparsity of microbiome data. These challenges have been addressed by using SpiecEasi [101], which covers these issues to a great extent and by calculating networks applying different filtering criteria. If the mediator is removed due to filtering, the data structure is affected [102]. As we interpreted only associations frequently detected across networks, this procedure reduces the odds of interpreting indirect associations.
4. Associations might be due to environmental conditions, without representing any interdependence relation among microbes [100]. Here, networks have been inferred from soil samples, which were comparable in physicochemical properties despite their different origins (Table 1). Consequently, the probability that environmental variables biased the networks, thereby introducing environmentally driven associations, is low. Naturally, unobserved variables may play an (unknown) role. Here, we were interested only in the differences in microbial associations comparing snow-free and snow covered soils. The comparability of site properties and compositions does not imply that the network predicted is complete or true, but it vouches for the comparability of the two networks, thereby allowing us to answer our questions.
5. Networks often assume static, not dynamic associations [103]. Here, we do not assume that microbes interact wherever and whenever they occur, because we did not sample an environmental gradient or inferred networks over time. Rather, we consider the samples as ecosystem representatives in the same state and we compared these states, which are overall comparable in composition. The variation modelled in the networks is therefore natural heterogeneity present in the ecosystem, which provides ecologically relevant variation. For this reason, there are no concerns regarding biases towards positive or negative associations, which may vary, depending on the ecological gradient observed [100].
6. Artificial filtering criteria was our biggest concern about the networks inferred. Due to the necessity of filtering prior to network inference [22, 23], networks will never give a complete representation of microbial associations. Therefore, there cannot be any claim on completeness. While we acknowledge that interpreting concrete microbial associations in more detail is laborious and might be biased, we believe that predicting and proposing them is valuable for future research and hypotheses, given that the associations proposed are well justified. Having the aim of investigating and proposing concrete microbial associations inferred from the analysis, we did not want them to depend on filtering criteria, which is always the case if only one filtering criterion is applied. We argue, that applying multiple filtering criteria and interpreting associations that are frequently found across criteria and involve taxa well-supported by the literature, allows for justified interpretation of concrete microbial associations. Therefore, we have achieved our aim of pinpointing possible associations typical for the habitat studied that can be the target of future research and validation. Since the study design has minimized the chances of false-positive associations and the analytical approach chosen allows for the interpretation of specific microbial associations, we believe that this design can be useful for studies in other ecosystems and beneficial for understanding microbial ecology.

As a last point, there is a controversy on analysing marker gene based microbiome data as ASVs, OTUs or zOTUs, especially with regards to fungal ITS amplicon sequencing ([104]). While this is a very relevant topic with far reaching impact, we do not want to add to this discussion. Here, we argue that our results and conclusions are largely unaffected from this controversy. Although we do discuss our results on genus and species level, none of our models has been calculated on genus or species level, i.e. we do not imply that ASVs (or OTUs or zOTUs) clearly delimit species. Our approach assumes that identical sequences come from similar or identical organisms, and that, in the best case, within-species sequence variation will correlate. On these reasonable assumptions, we interpreted correlation-based patterns composed of several ASVs with similar taxonomic annotation and ecological behaviour. We assume that the repeated observation inherent of these patterns is an indication for ecological signal, which is a commonly accepted assumption. While the need for repetition increases the stringency of our approach, it probably misses a number of ecological signals. From our perspective though, such loss does not cause any problems here, because we do not aim for completeness. In fact, we think this stringency adds to our approach, as a signal detected under these conditions is indeed a strong signal.

## Conclusions

Despite their geographical independency, the soil microbial community composition of several (sub-)alpine *P. cembra* forests was comparable. This is consistent with our expectations, as all forests shared similar climatic conditions and soil pH. Therefore, the fungal and bacterial core community, i.e. microbial units detected in all forests studied here, and their repeatedly found associations might present a typical community pattern for (sub-)alpine *P. cembra* forests. However, the current status might change due to environmental factors, e.g. higher temperatures or drought.

Contrary to our initial hypothesis, we did not observe a turnover of the entire microbial community caused by snow cover. Nevertheless, we observed increased microbial richness under snow cover compared to snow-free conditions, and we observed seasonal variations in the abundance and occurrence of specific taxa and microbial associations. The abundance and occurrence of specific taxa, such as *Russula, Suillus, Rhizopogon, Trechispora, Tetracladium*, and *Phenoliferia*, were found to depend on snow cover. This suggests their potential role in seasonal nutrient turnover. The networks inferred from snow covered soils were denser than those from snow-free soils and fungal ASVs demonstrated higher connectivity as bacterial ASVs. This highlights the role of fungi in complex nutrient cycling in coniferous forest soils. Seasonally dependent associations frequently involved *Mortierella* and mycorrhizal fungi, such as *Cladophialophora, Suillus,* and *Pseudotomentella.* This suggests that fungal bacterial associations might be closely linked to *P. cembra* root mycorrhization and its seasonality. Our study emphasises the intricacy of microbial interactions within a multi-level, complex context and urges for a comprehensive understanding of microbial ecology, which should include both mechanistic, direct interactions and ecosystem-level dynamics.

### Supplementary Information

**Additional file 1.** Supplementary figures.

**Additional file 2.** Supplementary tables.

## Data Availability

Illumina amplicon sequencing data are available in the NCBI SRA under the bioproject number PRJNA1005801. All code used for data analysis can be found on Github: Maraikep/Pinus. The supplementary information contains supporting information not presented in the main text.

## Abbreviations

ASV: Amplicon sequence variant
fb associations: Fungal-bacterial associations
a.s.l.: Above sea level
