# Supplementary material for: Fungal and bacterial communities and their associations in snow-free and snow covered (sub-)alpine Pinus cembra forest soils
Source: Environ Microbiome. 2024 Apr 2;19:20. doi: 10.1186/s40793-024-00564-7 (PMC10985912; doi:10.1186/s40793-024-00564-7)
Supplement: Supplementary file 1 — Additional file 1. Supplementary figures. [file 40793_2024_564_MOESM1_ESM.docx]

# Fungal and bacterial communities and their associations in snow-free and snow covered (sub-)alpine *Pinus cembra* forest soils

# Supplementary Information

Maraike Probst^1^, Anusha Telagathoti^1^, Edoardo Mandolini^1^, Ursula Peintner^1^

^1^ Universität Innsbruck, Austria; Department for Microbiology, Technikerstrasse 25, 6020 Innsbruck, Austria

*corresponding author

[Maraike.Probst@uibk.ac.at](mailto:Maraike.Probst@uibk.ac.at)

[Anusha.Telagathoti@uibk.ac.at](mailto:Anusha.Telagathoti@uibk.ac.at)

[Edoardo.Mandolini@uibk.ac.at](mailto:Edoardo.Mandolini@uibk.ac.at)

[Ursula.Peintner@uibk.ac.at](mailto:Ursula.Peintner@uibk.ac.at)


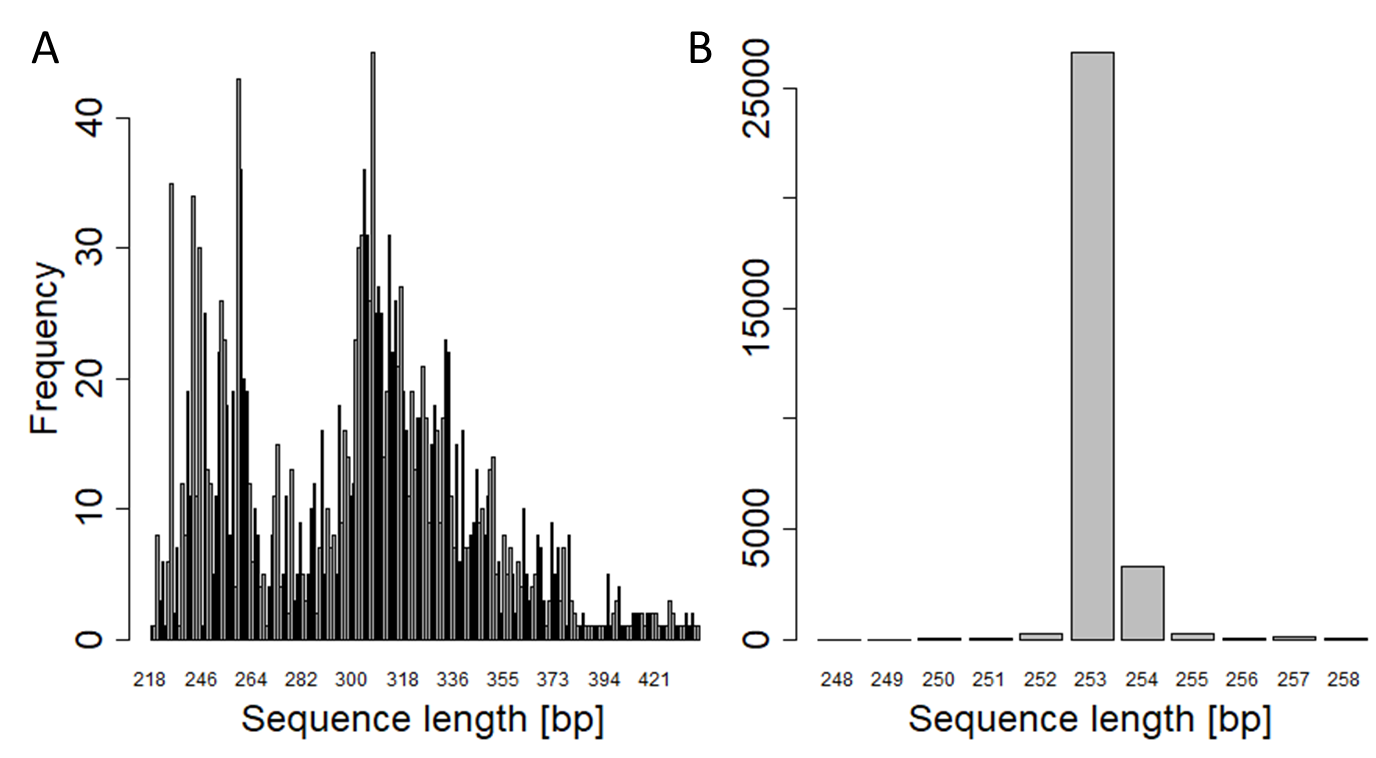


SI Fig. 1: Length distributions of fungal (A) and bacterial (B) ASV sequences.


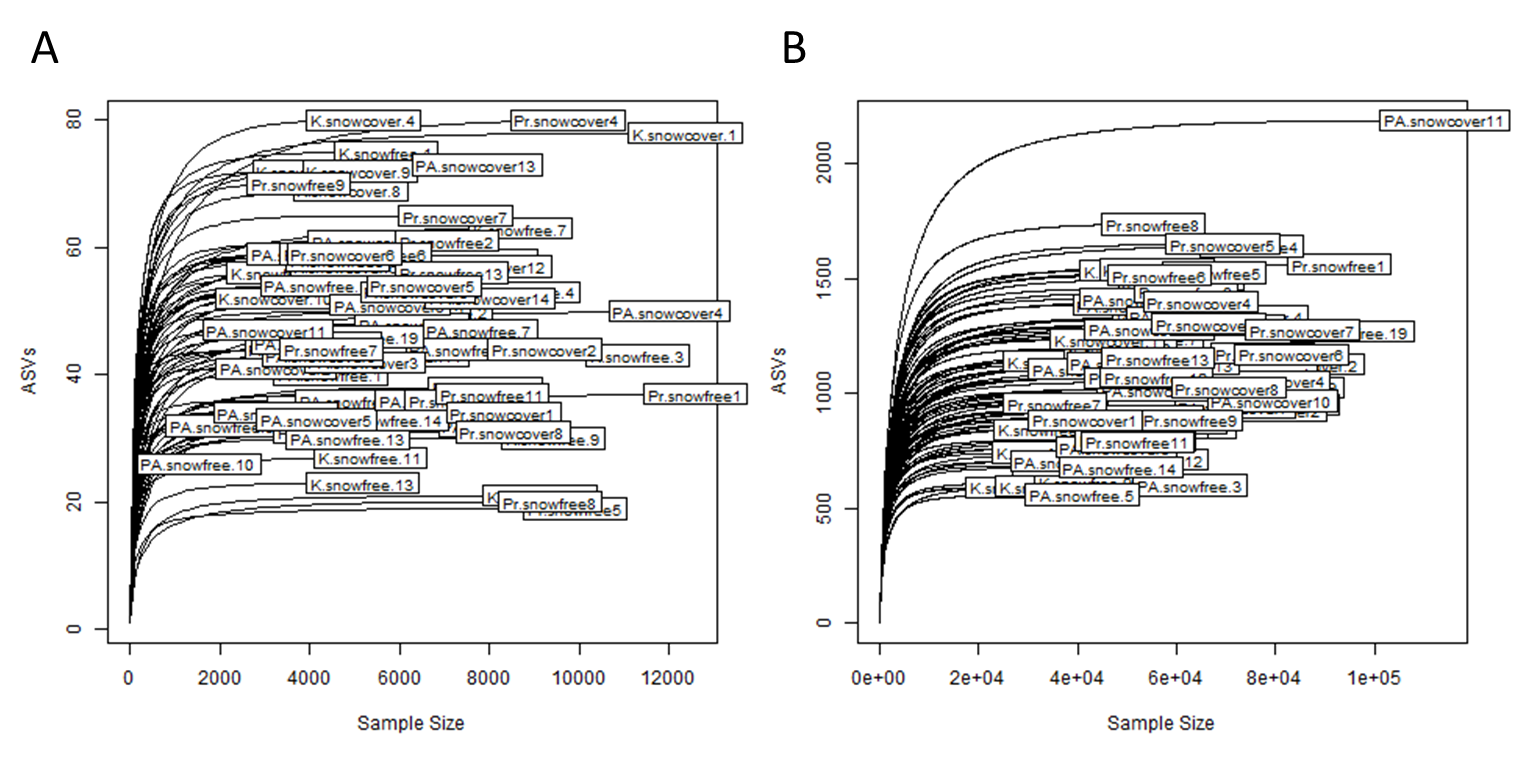


SI Fig. 2: Rarefaction curves from the (A) fungal and (B) bacterial dataset. K = Kühtai, PA = Patscherkofel, Pr = Praxmar.


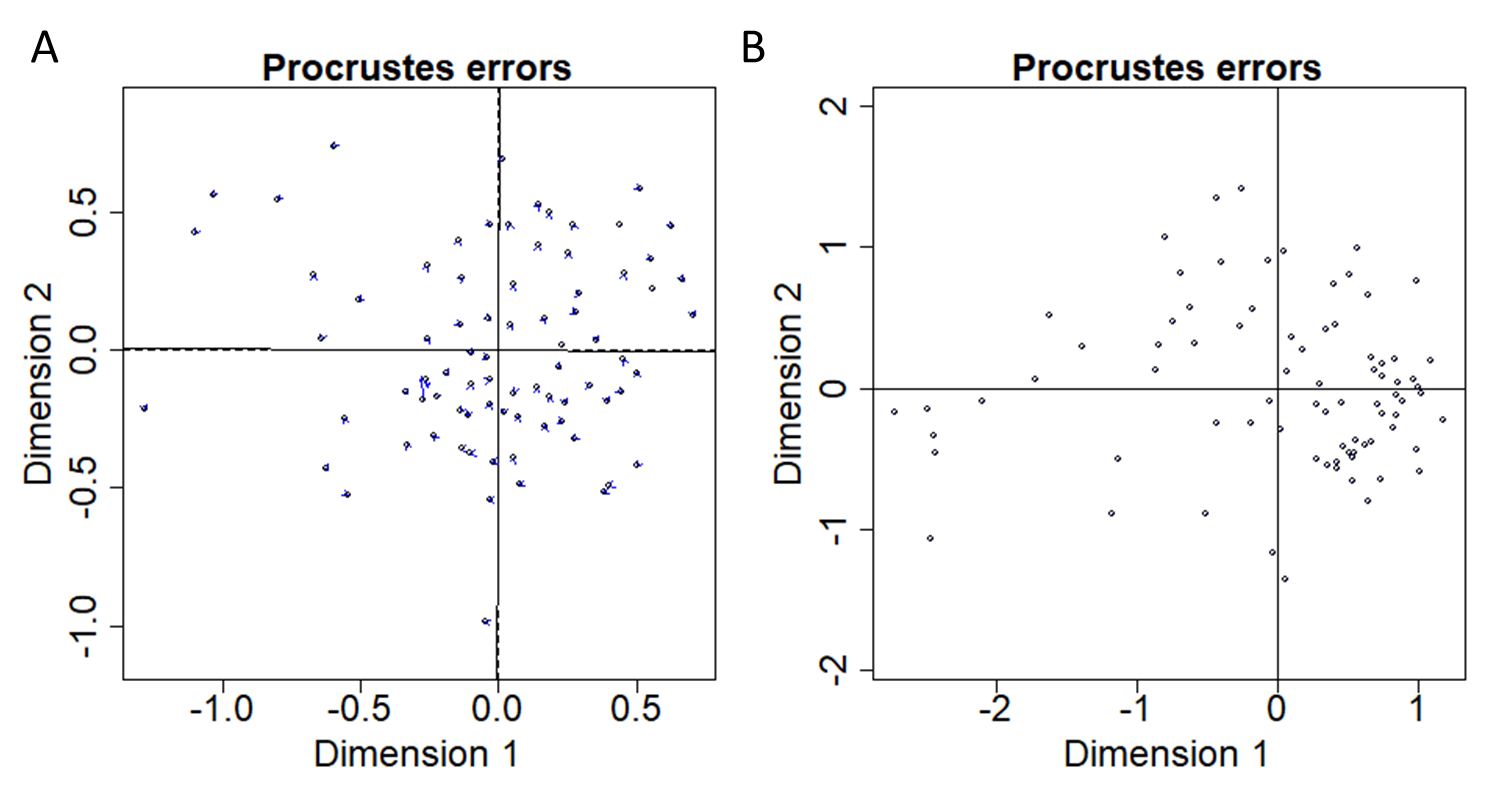


SI Fig. 3: Procrustes analysis comparing the rarefied and non-rarefied non-metric multidimensional scaling (NMDS) ordination based on Bray-Curtis similarity of the fungal (A) and bacterial (B) dataset.


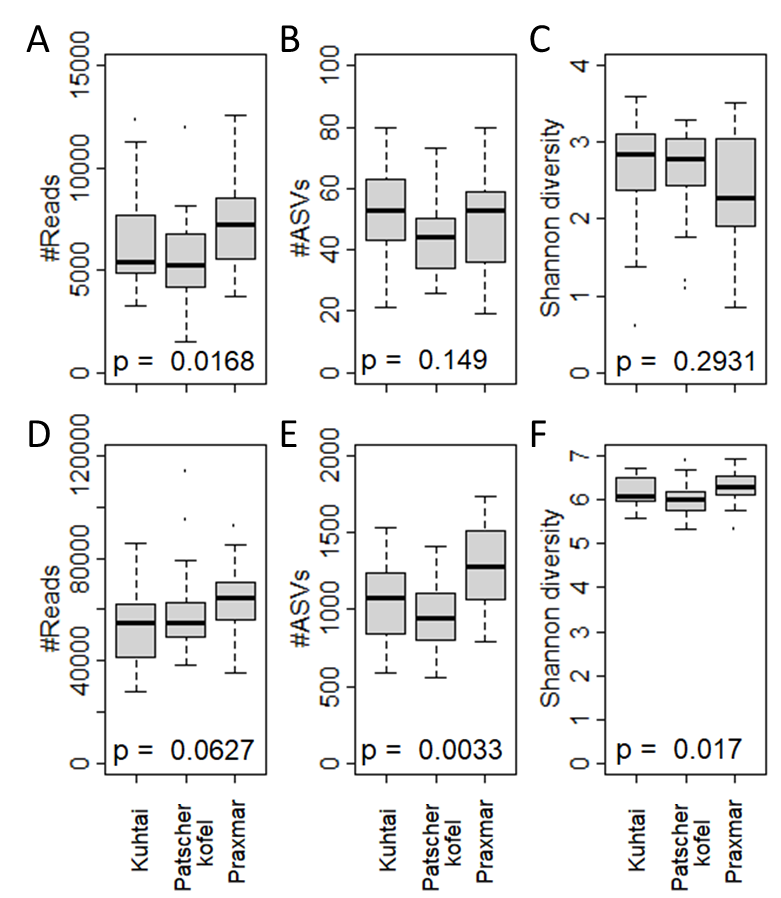


SI Fig. 4: Sequencing depth and alpha diversity of fungal (A-C) and bacterial (D-F) communities in sub-alpine *Pinus cembra* forests in different locations. (A, D) Sequencing depth of microbial communities collected. (B, E) Number of ASVs detected in the samples. (C, F) Shannon diversity index of the microbial communities.


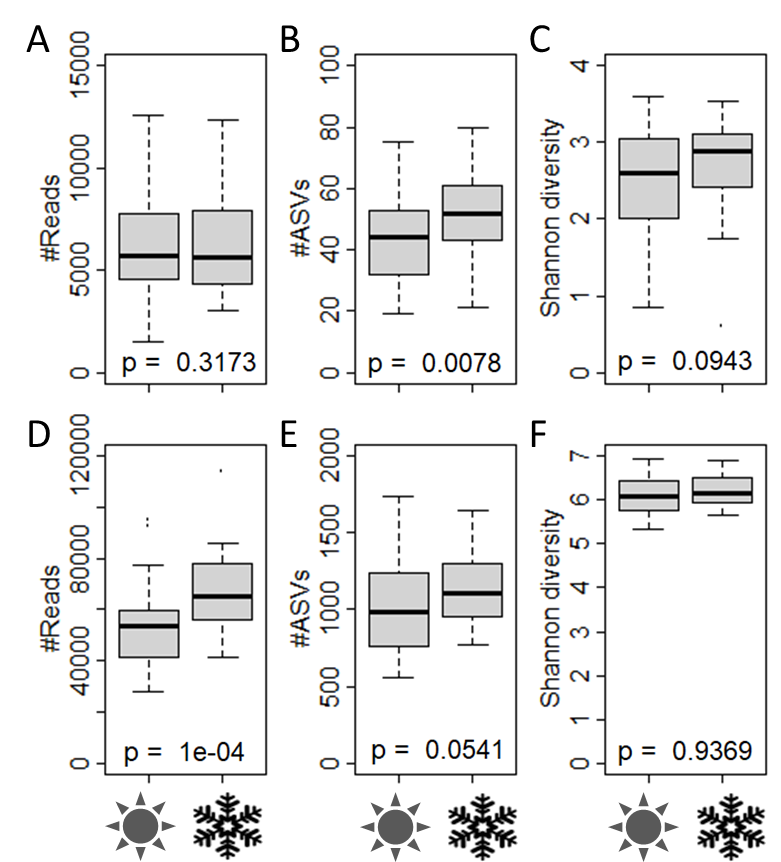


SI Fig. 5: Sequencing depth and alpha diversity of fungal (A-C) and bacterial (D-F) communities in sub-alpine *Pinus cembra* forest in snow-free and snow covered soils. (A, D) Sequencing depth of microbial communities collected. (B, E) Number of ASVs detected in the samples. (C, F) Shannon diversity index of the microbial communities.


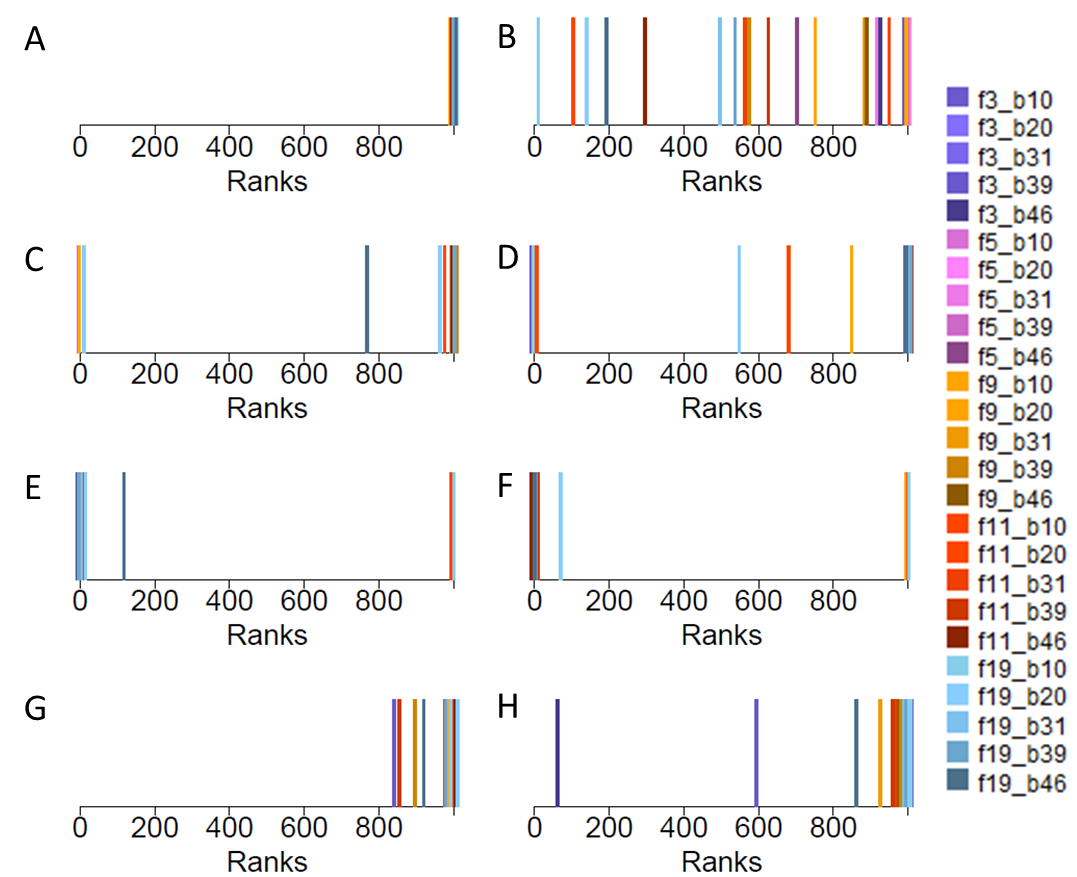


SI Fig. 6: Comparison between observed network statistics and random pickings. For each bacterial and fungal association network inferred, 999 comparable random association networks were inferred. This was achieved by drawing the same number of associations as inferred by each network from the same number of different fungal and bacterial ASVs, respectively, as used for network inference. The numbers of (A) shared associations and (B) shared fungal and bacterial associations were counted for all, inferred and random, networks. Moreover, the frequencies in both snow-free and snow covered networks of (C, D) fungal-fungal, (E, F) fungal-bacterial, (G, H) bacterial-bacterial associations were counted for all, inferred and random, networks. The rank of the inferred network within each respective random distribution is indicated. In other words: Network traits with ranks between 950-1000 are significantly higher in the inferred network compared to random pickings; network traits with ranks between 0-50 are significantly lower in the inferred network compared to random pickings.


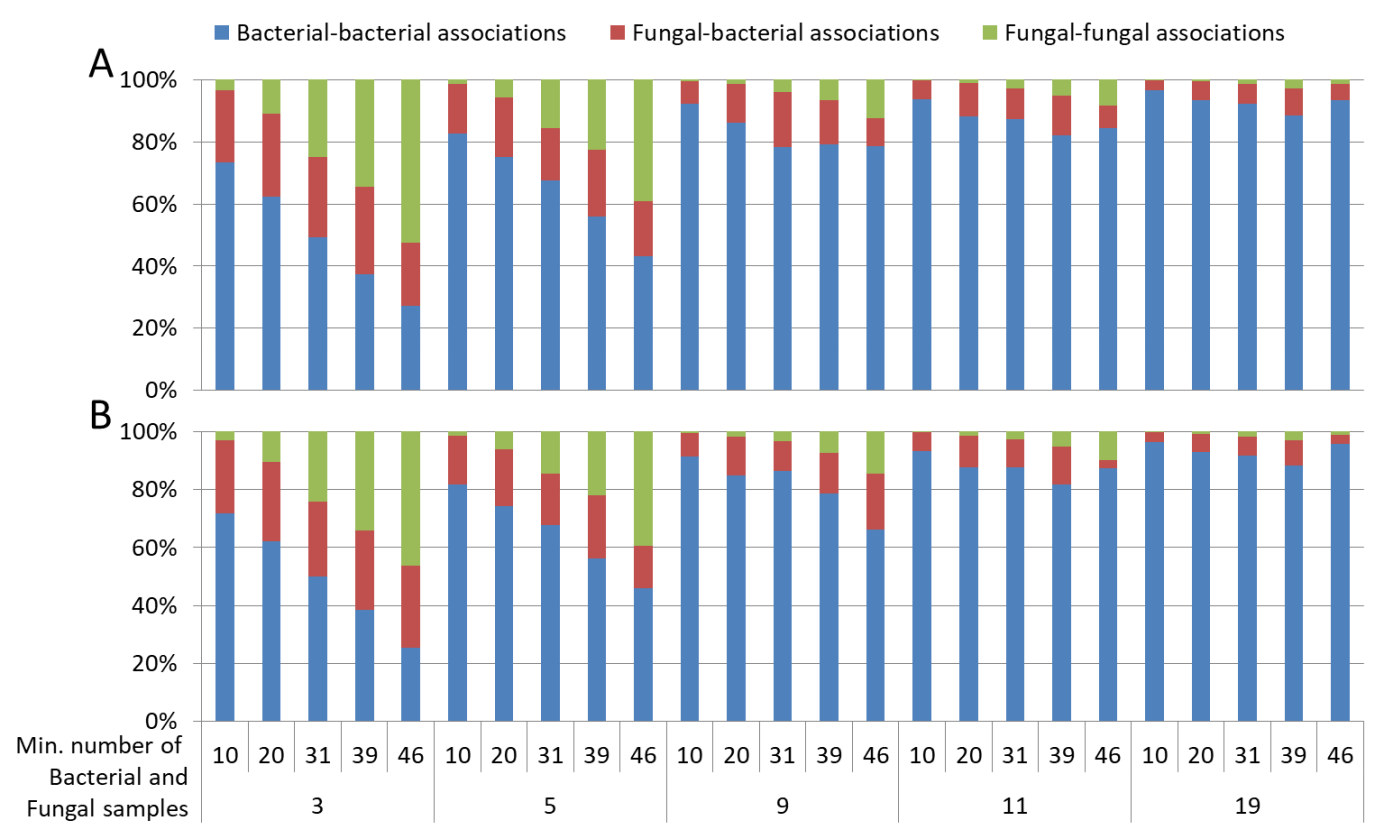


SI Fig. 7:Proportions of bacterial-bacterial, fungal-bacterial and fungal-fungal associations in networks inferred for (A) snow-free and (B) snow covered *P. cembra* forests.


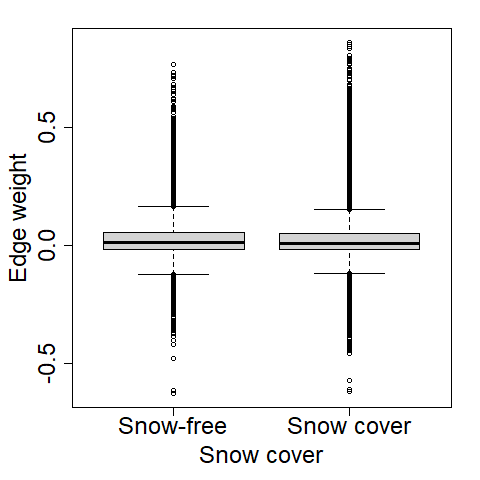


SI Fig. 8: Edge weight distribution across all snow-free and snow covered networks.


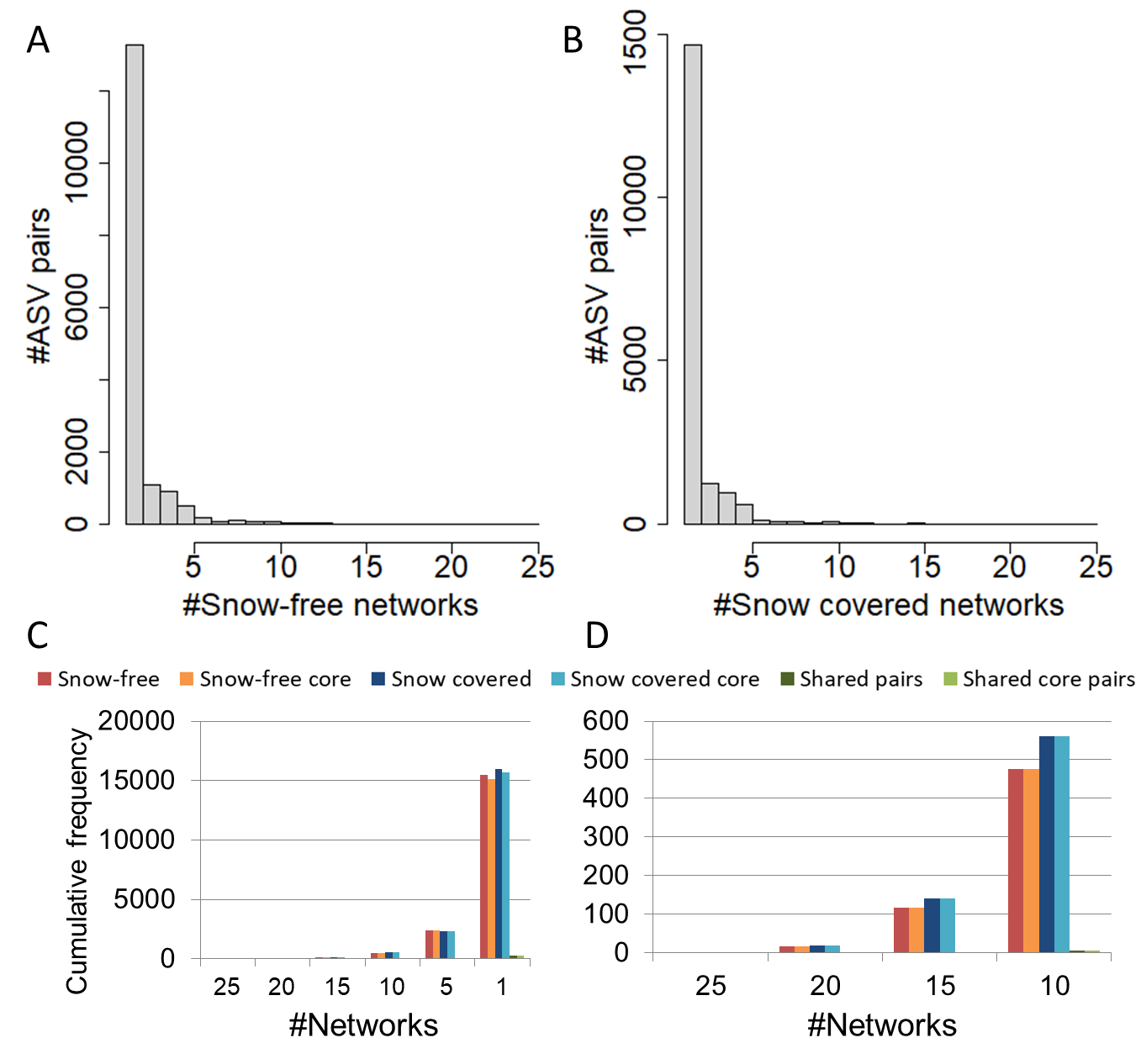


SI Fig. 9: Distribution of ASV associations across networks. The frequency of ASV associations detected in and across (A) snow-free and (B) snow covered networks is illustrated. (C, D) The cumulative number of ASV associations detected at different thresholds. Panel (D) zooms into panel (C).
